# *Carpobrotus edulis* Flower Extract Exerts Cytotoxic and Apoptotic Effects on A-549 Lung Cancer Cells: Phytochemical Profiling and In Silico EGFR Interaction Screening

**DOI:** 10.3390/ijms272110016

**Published:** 2026-11-08

**Authors:** Bader Huwaimel, Amr S. Abouzied, Marwa M. Abd Rabo, Mohamed A. Said, Saad Alqarni, Ali Alghubayshi, Hashem O. Alsaab, Rami M. Alzhrani, Wejdan S. Alshehri, Nouf Eid Alrashidi, Lamya A. Alwahdani, Kareem M. Younes, Mohamed S. Refaey

**Affiliations:** 1 Department of Pharmaceutical Chemistry, College of Pharmacy, University of Hail, Hail 81442, Saudi Arabia; b.huwaimel@uoh.edu.sa (B.H.); s.alqarni@uoh.edu.sa (S.A.); k.younes@uoh.edu.sa (K.M.Y.); 2 Medical and Diagnostic Research Centre, University of Hail, Hail 55473, Saudi Arabia; a.alghubyshi@uoh.edu.sa; 3 Department of Biochemistry, Egyptian Drug Authority (Formerly National Organization for Drug Control and Research), Giza 12553, Egypt; marwa10mokhtar1@outlook.com; 4 Department of Pharmaceutical Chemistry, Faculty of Pharmacy, Egyptian Russian University, Cairo 11829, Egypt; mohamed-adel@eru.edu.eg; 5 Drug Design and Discovery Laboratory, Helmy Institute for Medical Sciences, Zewail City of Science and Technology, 6th of October City 12578, Egypt; 6 Department of Clinical Pharmacy, College of Pharmacy, University of Hail, Hail 81442, Saudi Arabia; 7 Department of Pharmaceutics and Industrial Pharmacy, College of Pharmacy, Taif University, P.O. Box 11099, Taif 21944, Saudi Arabia; h.alsaab@tu.edu.sa (H.O.A.); r.zhrani@tu.edu.sa (R.M.A.); 8 College of Pharmacy, University of Hail, Hail 81442, Saudi Arabia; alseds.wejdan@gmail.com (W.S.A.); alkiari.nouf@gmail.com (N.E.A.); alenazi.lamya@gmail.com (L.A.A.); 9 Department of Pharmacognosy, Faculty of Pharmacy, University of Sadat City, Sadat City 32897, Egypt; 10 Faculty of Pharmacy, National University of Sadat City, Sadat City 32897, Egypt

**Keywords:** *Carpobrotus edulis*, cytotoxicity, GC-MS analysis, molecular dynamics, EGFR kinase

## Abstract

Medicinal plants serve as critical reservoirs of bioactive scaffolds for targeted cancer therapeutics. This study evaluated the antiproliferative potential of *Carpobrotus edulis* methanolic flower extract across a panel of human cancer cell lines, including pancreatic (Panc-1), breast (MCF-7), liver (HepG-2), and lung (A-549) malignancies. Screening assays revealed potent, dose-dependent cytotoxicity in an MTT-based viability assay, with A-549 lung cancer cells being the most sensitive of the four tested cancer cell lines (IC_50_ = 3.62 ± 0.34 μg/mL). Selectivity over non-malignant cells was not assessed. Flow cytometry showed an increase in the S-phase fraction and in Annexin V-positive cells, and ELISA showed higher Bax and p53 and lower Bcl-2 in extract-treated A-549 cells, consistent with apoptosis through the intrinsic pathway. The measured caspase-3 concentration was lower after treatment, and the assay does not distinguish pro-caspase-3 from cleaved caspase-3. Phytochemical profiling via GC-MS identified a diverse array of bioactive metabolites, predominantly triterpenoids, phytosterols, fatty acid derivatives, and cyclitols. To explore EGFR as a putative target, an in vitro EGFR kinase assay was combined with molecular docking and molecular dynamics (MD) simulations. Among the profiled metabolites, the cyclitol derivative mome inositol displayed the most favorable docking score binding affinity toward the EGFR kinase domain (−7.35 kcal/mol), establishing robust hydrogen-bonding networks with key catalytic residues within the active site. Furthermore, a 100 ns MD simulation revealed the relative thermodynamic stability of the mome inositol–EGFR complex with more favorable RMSD, RMSF, hydrogen bond and radius of gyration profiles than the co-crystallized reference ligand. Collectively, these integrated in vitro, phytochemical, and in silico findings indicate that methanolic extract of *C. edulis* flowers exerts potent anticancer activity predominantly through mitochondrial-mediated apoptosis complemented by a computationally predicted interaction between its major cyclitol constituent and the EGFR kinase domain. These results establish the extract as a promising early-stage anticancer lead and provide a clear rationale for advancing to mechanistic studies, with in vivo validation reserved for subsequent investigations.

## 1. Introduction

Cancer remains a major global health challenge, with epidemiological projections indicating that the annual burden of new cases may surpass 35 million by 2050 [1]. Although conventional treatment modalities such as systemic chemotherapy remain cornerstone strategies in oncological management, their clinical utility is severely limited by narrow therapeutic indices and profound adverse effects, including cardiotoxicity, neurotoxicity, and myelosuppression [1,2,3]. These clinical limitations have intensified the search for safer, more selective therapeutic alternatives, particularly those derived from medicinal plants. Phytochemical scaffolds optimized by nature represent an invaluable reservoir of structurally diverse lead compounds possessing multi-targeted anticancer properties. *Carpobrotus edulis* (*C. edulis*), a succulent plant belonging to the Aizoaceae family, is traditionally used in various regions for its medicinal benefits. Phytochemical investigations have revealed that its vegetative tissues—particularly the leaves—are rich in flavonoids, tannins, alkaloids, and phenolic acids, compounds widely recognized for their antioxidant, anti-inflammatory, antimicrobial, and cytoprotective activities [4,5,6,7,8,9]. Emerging evidence also highlights the anticancer potential of *C. edulis* extracts. Previous studies have largely focused on leaf-derived extracts and have demonstrated multiple anticancer-related mechanisms across different cancer models. Methanolic extracts and isolated constituents inhibited P-glycoprotein-mediated efflux in multidrug-resistant mouse lymphoma cells [10], while a 70% methanolic leaf extract suppressed multidrug-resistance efflux-pump activity and enhanced the killing of phagocytosed *S. aureus* in L5178 mouse T-cell lymphoma cells [11]. Furthermore, a hydroethanolic leaf extract reduced the viability of HCT-116 human colon cancer cells and exhibited antiglycation and antioxidant activities [12]. Aqueous extracts displayed cytotoxic effects against U251 and U87 glioblastoma cells and induced ROS-mediated apoptosis in SK-N-BE(2) and SH-SY5Y neuroblastoma cells, as evidenced by increased caspase-9 activity, mitochondrial membrane depolarization, and elevated ROS production [13,14]. More recently, methanol and hexane leaf extracts were reported to induce apoptotic morphological changes and inhibit migration in ME-180 cervical and A375 melanoma cell lines, while showing no adverse effects on a normal cell line [15]. In contrast, studies involving *C. edulis* flowers remain limited and have primarily focused on phytochemical composition and antioxidant or antimicrobial properties. Notably, flowers were reported to possess the highest phenolic content and the strongest antioxidant activity among the plant parts investigated [9,16]. To the best of our knowledge, despite the growing body of evidence regarding the biological activities of *C. edulis*, the anticancer potential of its flower extracts has not been comprehensively investigated. This knowledge gap prompted the present study to evaluate the phytochemical profile and anticancer activity of *C. edulis* methanolic flower extract.

A critical node in modern targeted oncology is the Epidermal Growth Factor Receptor (EGFR), a transmembrane receptor tyrosine kinase that governs essential cellular networks, including proliferation, survival, migration, and angiogenesis. Aberrant EGFR expression, amplification, or hyperactivation is a defining hallmark of non-small cell lung cancer (NSCLC), closely correlating with poor clinical prognosis, accelerated metastasis, and resistance to conventional chemo- and radiotherapy [17]. Consequently, the EGFR kinase domain has emerged as a major target for small-molecule intervention. While synthetic tyrosine kinase inhibitors (TKIs) are clinically available, emerging secondary mutations and systemic toxicities necessitate the continuous discovery of novel, naturally derived EGFR inhibitors.

Given the reported bioactivities of *C. edulis* and the therapeutic relevance of EGFR inhibition, this study examined the antiproliferative effects of a methanolic extract of *C. edulis* flowers in four human cancer cell lines, with particular attention to A-549 lung cancer cells and to apoptosis-related changes in these cells. EGFR was investigated as a plausible, literature-supported candidate target, using an in vitro kinase assay, GC-MS-guided docking and molecular dynamics simulations. By integrating in vitro enzymatic assays with advanced in silico molecular docking and 100 ns molecular dynamics simulations, this work seeks to provide a hypothesis-generating mechanistic rationale, rather than definitive proof, for the use of *C. edulis* flower-derived metabolites as candidate leads in lung oncology.

## 2. Results

### 2.1. Cytotoxic Activity

**Figure 1:**
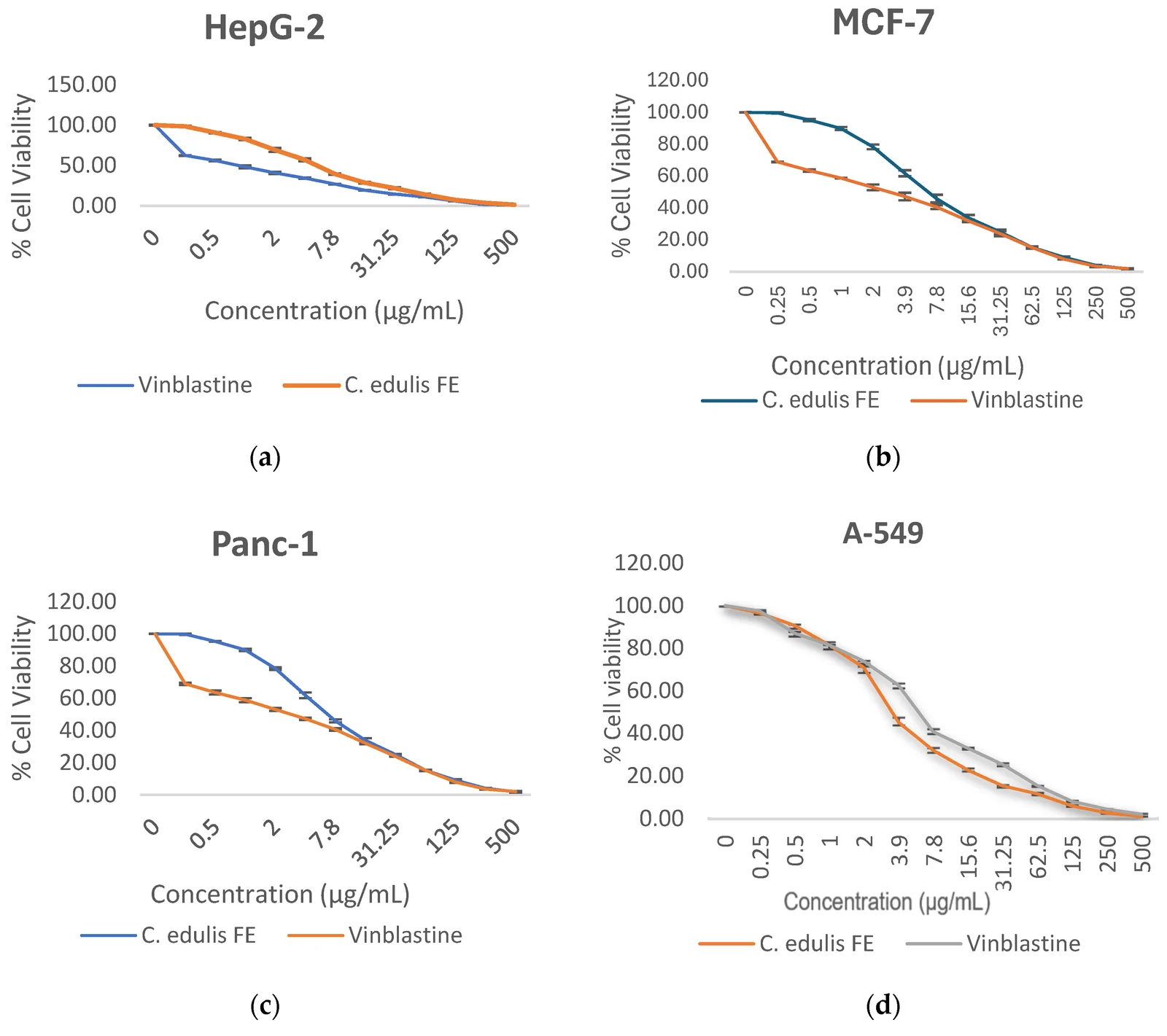
Dose-dependent viability: (**a**) HepG-2 cell line. (**b**) Panc-1 cell line. (**c**) MCF-7 cell line and (**d**) A-549 cell line after treatment with the methanolic flower extract of *C. edulis*.

The antiproliferative potential of *Carpobrotus edulis* methanolic flower extract was systematically screened against a panel of four human cancer cell lines—HepG-2, MCF-7, Panc-1, and A-549—using the MTT viability assay (Table 1, Figure 1).

The flower extract demonstrated potent, dose-dependent cytotoxicity across all tested lines. Among the evaluated cell lines, the lowest IC_50_ was observed in A-549 non-small cell lung cancer cells (3.62 ± 0.34 µg/mL). Panc-1 and HepG-2 cells showed moderate sensitivity, with IC_50_ values of 4.17 ± 0.32 µg/mL and 4.86 ± 0.48 µg/mL, respectively, whereas MCF-7 breast cancer cells proved to be the least sensitive (IC_50_ = 6.79 ± 0.45 µg/mL).

In comparison, the reference chemotherapeutic agent vinblastine sulfate exhibited IC_50_ values of 6.14 ± 0.43 µg/mL (A-549), 1.67 ± 0.19 µg/mL (Panc-1), 0.89 ± 0.13 µg/mL (HepG-2), and 3.02 ± 0.26 µg/mL (MCF-7). The crude flower extract demonstrated a significantly lower IC_50_ value than vinblastine in A-549 cells (*p* < 0.05), indicating greater cytotoxic potency under the experimental conditions employed. However, because the study did not include a non-cancerous control cell line, these findings should not be interpreted as evidence of tumor-selective cytotoxicity.

### 2.2. Antioxidant Activity

**Figure 2:**
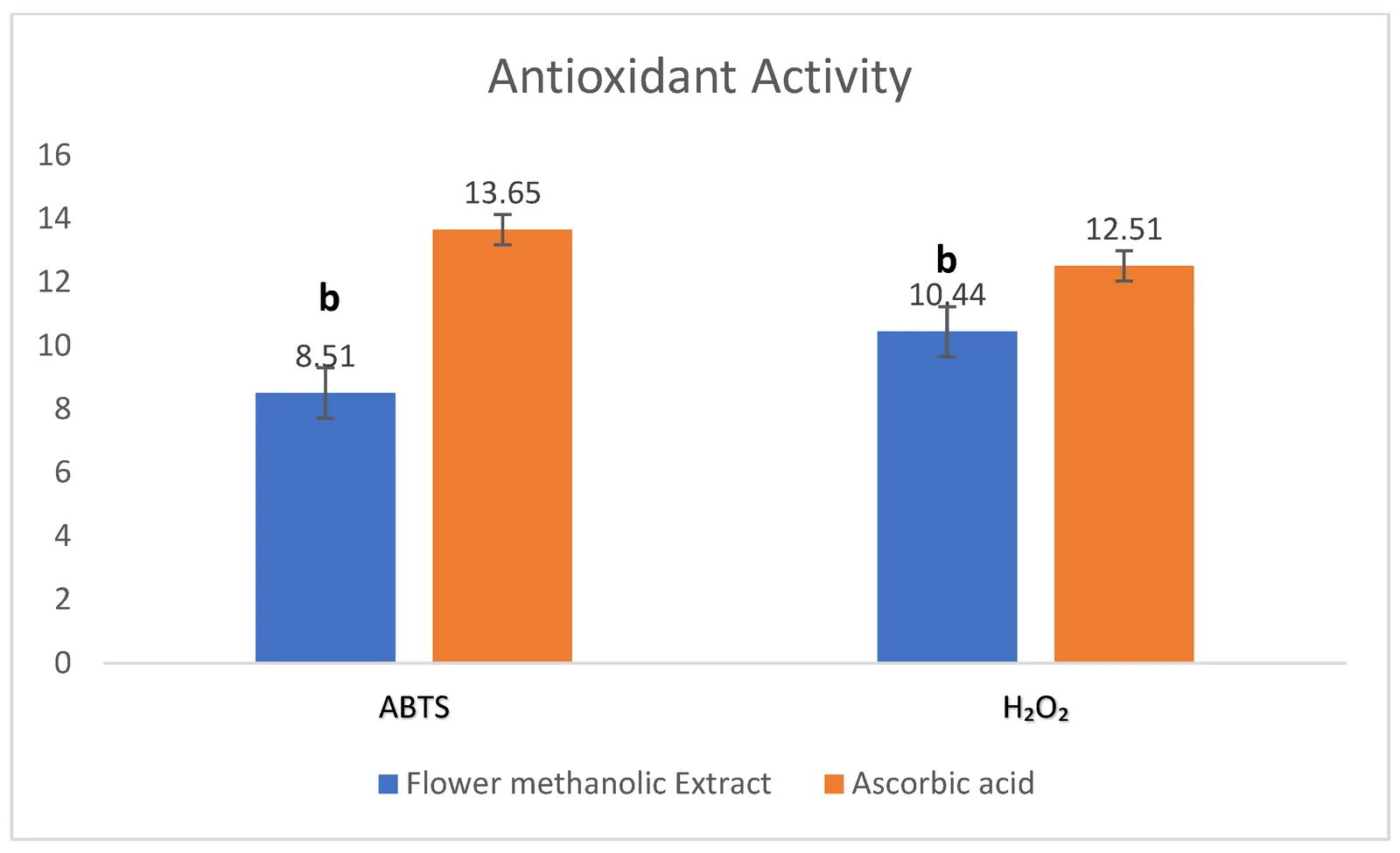
IC_50_ of *C. edulis* flower methanolic extract and ascorbic acid. The data, obtained from three replicates, are stated as mean ± SD, as (b) *p* < 0.05, related to the ascorbic acid group. The sample (blue) and the standard (orange).

To evaluate whether the cytotoxic footprint correlates with antioxidant defenses, the radical-scavenging properties of the flower extract were determined using parallel ABTS and H_2_O_2_ assays (Table 2, Figure 2). The extract exhibited robust, concentration-dependent antioxidant capacity, yielding IC_50_ values of 8.51 ± 0.79 µg/mL against (ABTS) radicals and 10.44 ± 2.19 µg/mL for (H_2_O_2_) neutralization. Crucially, these values were significantly lower than those recorded for the standard antioxidant ascorbic acid IC_50_ values of 13.65 ± 0.48 µg/mL (ABTS) and 12.51 ± 0.56 µg/mL (H_2_O_2_), establishing the superior radical-scavenging efficiency of the floral metabolites.

### 2.3. Cell Cycle Distribution

**Figure 3:**
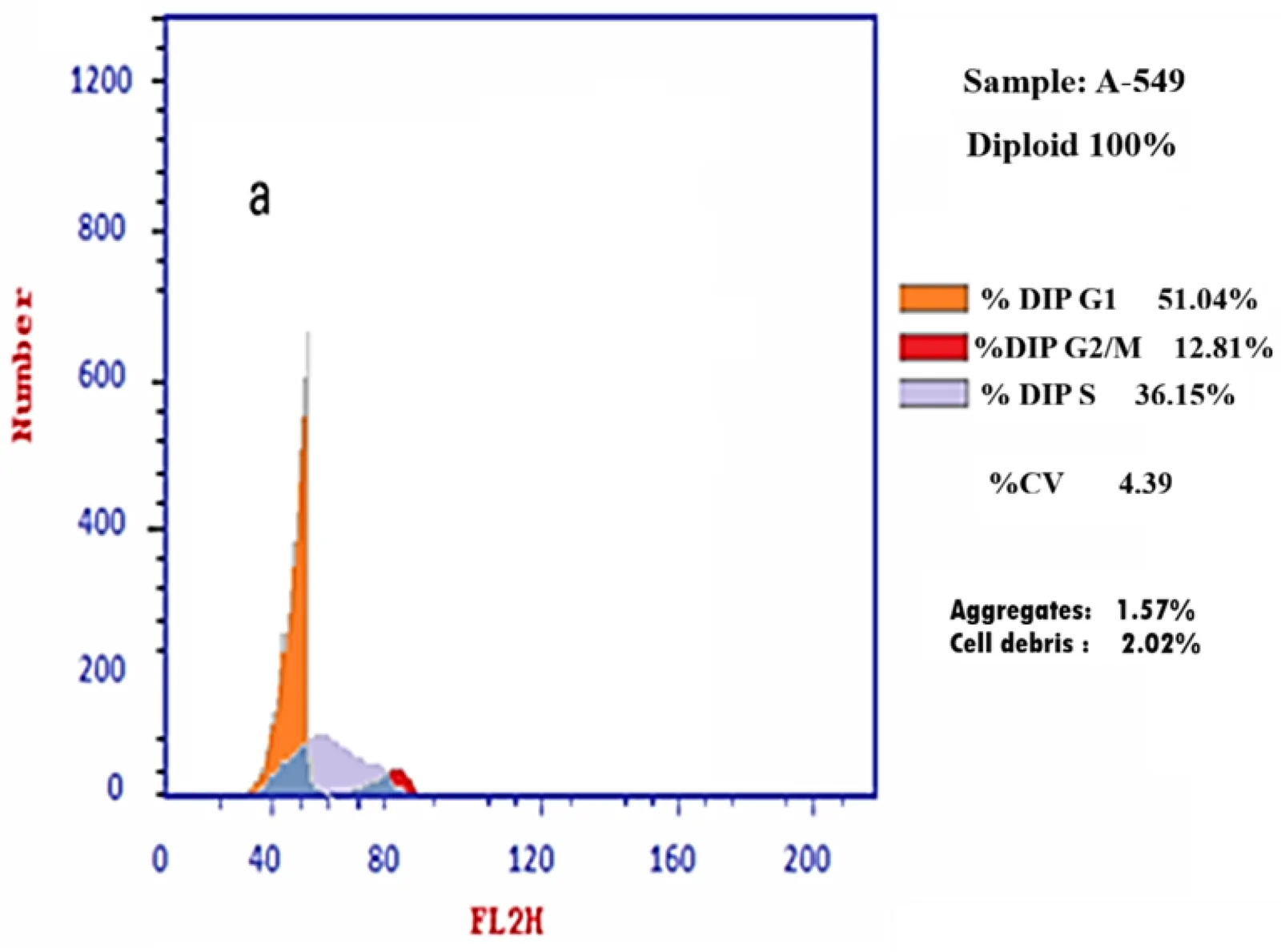

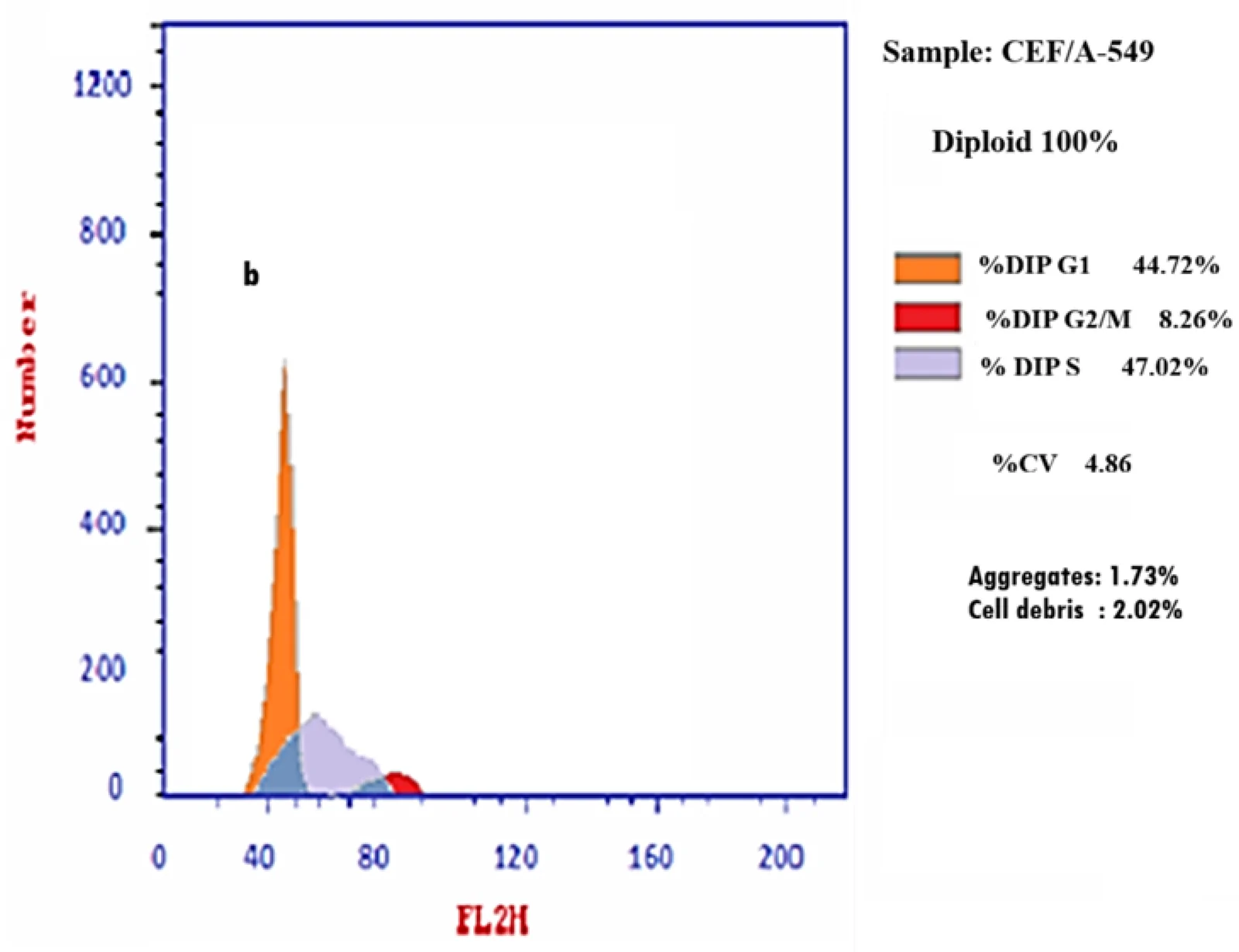
Cell-cycle distribution of A-549 lung cancer cells following treatment with *Carpobrotus edulis* flower methanolic extract. DNA content analysis was performed by flow cytometry after 72 h exposure to the IC_50_ concentration (3.62 µg/mL). (**a**) Histogram of untreated A-549 cells showing normal cell-cycle phase distribution. (**b**) Histogram of extract-treated A-549 cells demonstrating alterations in cell-cycle progression compared with control.

To elucidate the cell cycle dynamics underlying the observed antiproliferative effects, flow cytometry was performed on A-549 cells treated with the extract at its IC_50_ (3.62 µg/mL). Exposure to the extract induced a prominent, statistically significant redistribution of cell-cycle phases, with a marked increase in the S-phase population. Specifically, the S-phase fraction increased from 36.43% in untreated cells to 45.26% in treated cells (*p* = 0.004) (Table 3, Figure 3a,b). These findings indicate S-phase accumulation and suggest that the extract influences cell-cycle progression in A-549 cells.

### 2.4. Apoptosis Induction

**Figure 4:**
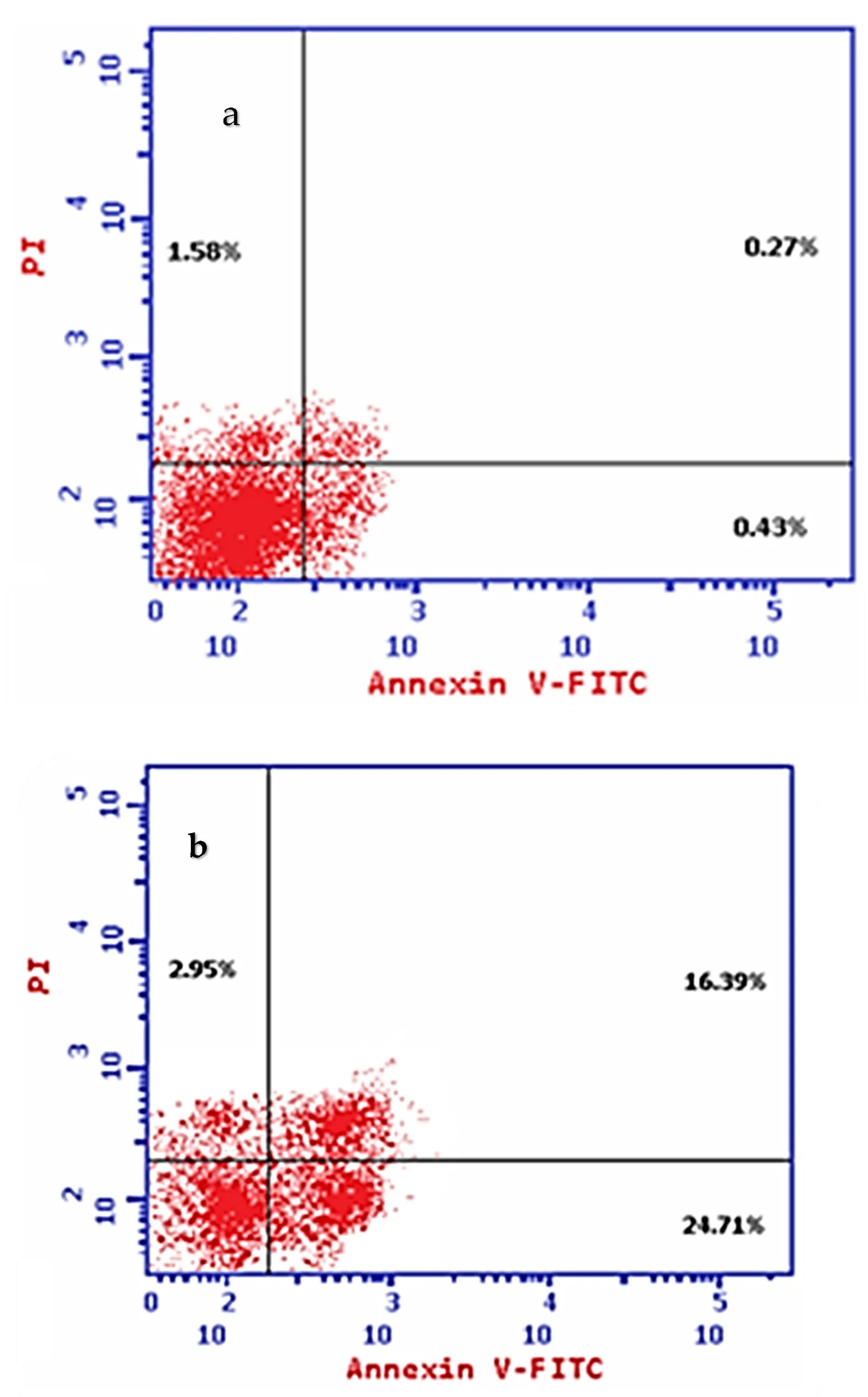
Annexin V/PI apoptosis analysis of A-549 lung cancer cells following treatment with *Carpobrotus edulis* flower methanolic extract. Cells were exposed to the IC_50_ concentration for 72 h and subsequently stained with Annexin V-FITC and propidium iodide to assess apoptotic and necrotic populations. (**a**) Untreated A-549 cells showing predominantly viable cell populations. (**b**) Extract-treated A-549 cells demonstrating increased early and late apoptotic populations compared with control, indicating extract-induced apoptosis.

To distinguish between programmed cell death and passive necrosis, treated A-549 cells were analyzed using Annexin V-FITC/PI double staining. The extract provoked an increase toward apoptotic populations; early apoptotic cells increased dramatically from 0.43% (control) to 24.71% (treated), while late apoptotic cells rose from 0.27% to 16.39% (Figure 4a,b). Conversely, the necrotic cell fraction remained minimal, changing only slightly from 1.58% to 2.95%. These quantitative data verify that the primary cytocidal mechanism of the extract is governed by apoptosis rather than unprogrammed necrosis.

### 2.5. Modulation of Apoptosis-Related Biomarkers

To elucidate the molecular events associated with extract-induced apoptosis, the expression levels of key apoptosis-related markers were quantified in A-549 cells using ELISA (Table 4). Treatment at the IC_50_ concentration resulted in increased levels of the tumor suppressor protein p53 and the pro-apoptotic protein Bax, accompanied by suppression of the anti-apoptotic protein Bcl-2. These changes led to a marked elevation in the Bax/Bcl-2 ratio, supporting activation of the mitochondrial apoptotic pathway. In contrast, a reduction in the measured caspase-3 concentration was observed following treatment. Together with the Annexin V-FITC/PI apoptosis analysis, these findings indicate that the *C. edulis* flower methanolic extract promotes apoptotic cell death in A-549 cells.

### 2.6. In Vitro EGFR Kinase Inhibition

**Figure 5:**
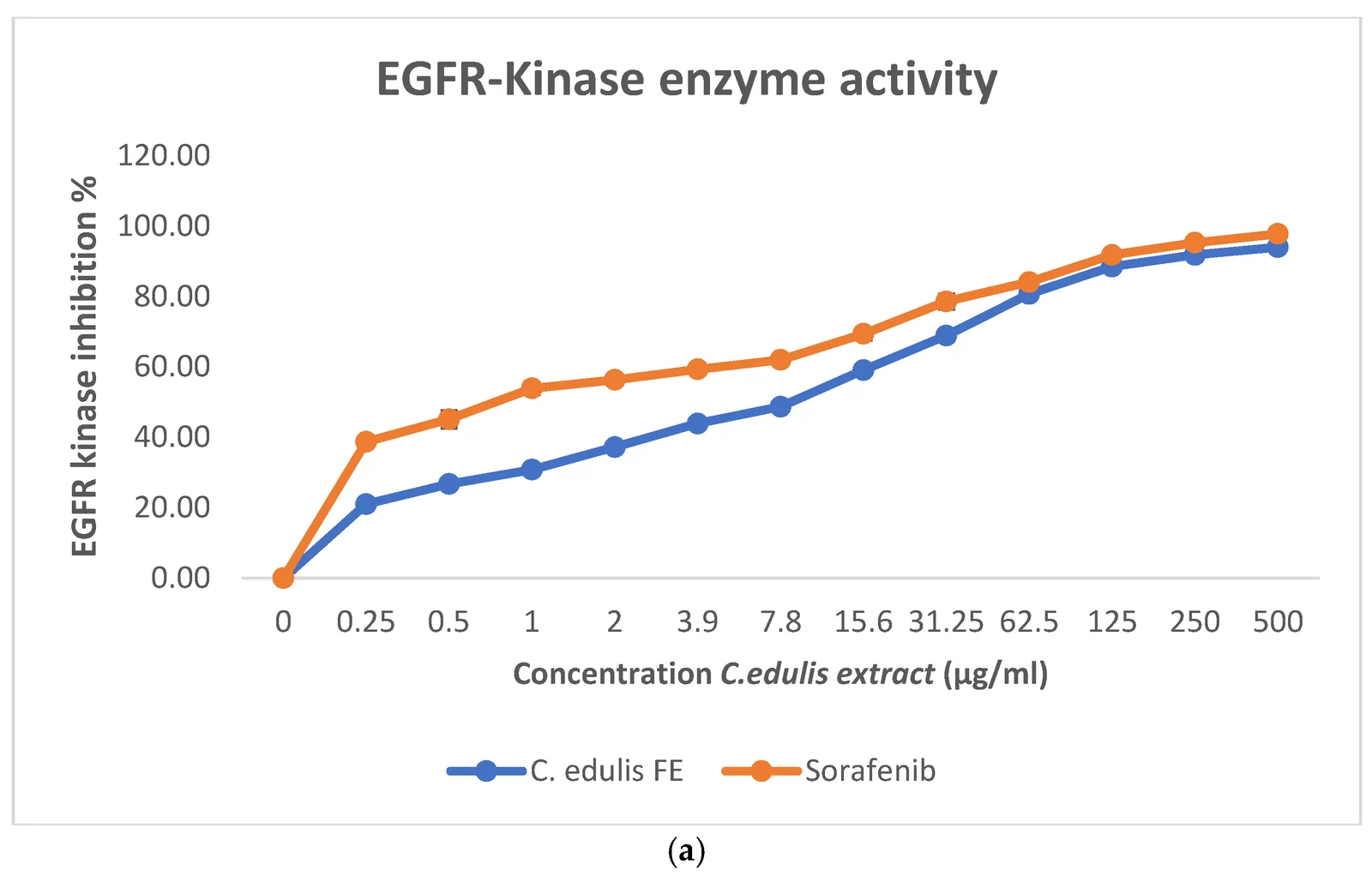

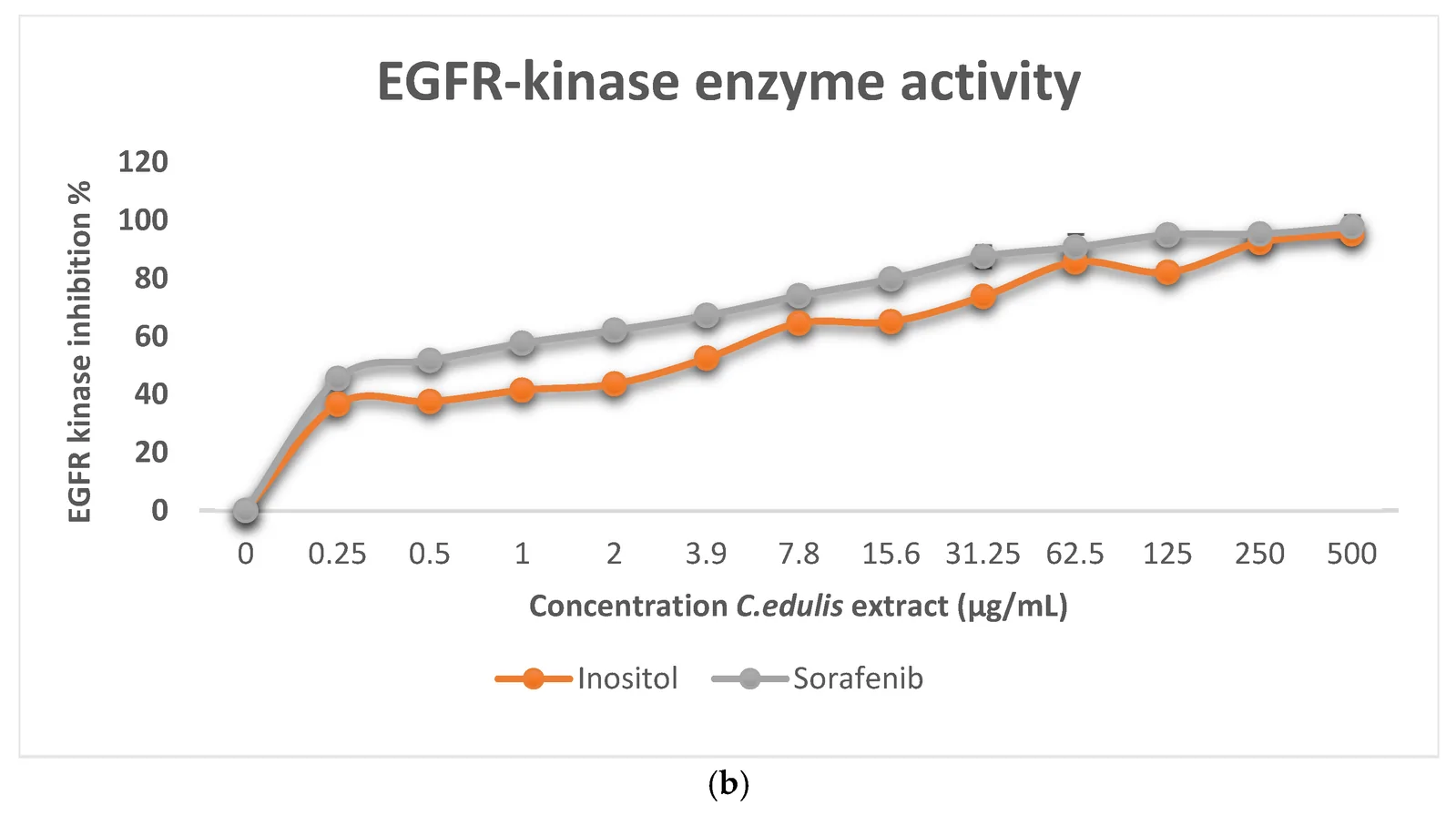
(**a**) In vitro kinase inhibitory activity of *C. edulis* flower methanolic extract and Sorafenib against EGFR kinase. Results are expressed as the mean and SD of triplicate experiments. (**b**) In vitro kinase inhibitory activity of mome inositol and Sorafenib against EGFR kinase. Results are expressed as the mean and SD of triplicate experiments.

Given that A-549 was the most sensitive of the four cell lines, the direct inhibitory impact of the extract and the identified mome inositol compound using GC-MS analysis on the Epidermal Growth Factor Receptor (EGFR) was measured via an in vitro enzymatic kinase assay (Figure 5a,b). The flower extract as well as mome inositol effectively suppressed EGFR kinase activity in a cell-free assay, with IC_50_ values of 4.75 ± 0.63 µg/mL for the flower extract and 2.9 ± 0.23 µg/mL for mome inositol, using sorafenib (IC_50_ value of 0.77 ± 0.15 µg/mL) as a reference multi-kinase inhibitor control. These findings showed that the extract and the identified mome inositol compound can inhibit purified EGFR enzymatic activity in a cell-free system; however, on their own, they do not establish that EGFR inhibition is the operative mechanism behind the extract’s cytotoxicity in A-549 cells, which carry a KRAS G12S mutation known to uncouple proliferation from EGFR signaling. Accordingly, the EGFR kinase assay and docking results are interpreted as supportive mechanistic indicators rather than definitive evidence of EGFR-dependent cytotoxicity. Direct cellular target-engagement studies (e.g., phospho-EGFR Western blotting or phospho-RTK profiling in extract-treated A-549 cells) are required to test this link conclusively and are identified as a priority for future work.

### 2.7. GC–MS Analysis

**Figure 6:**
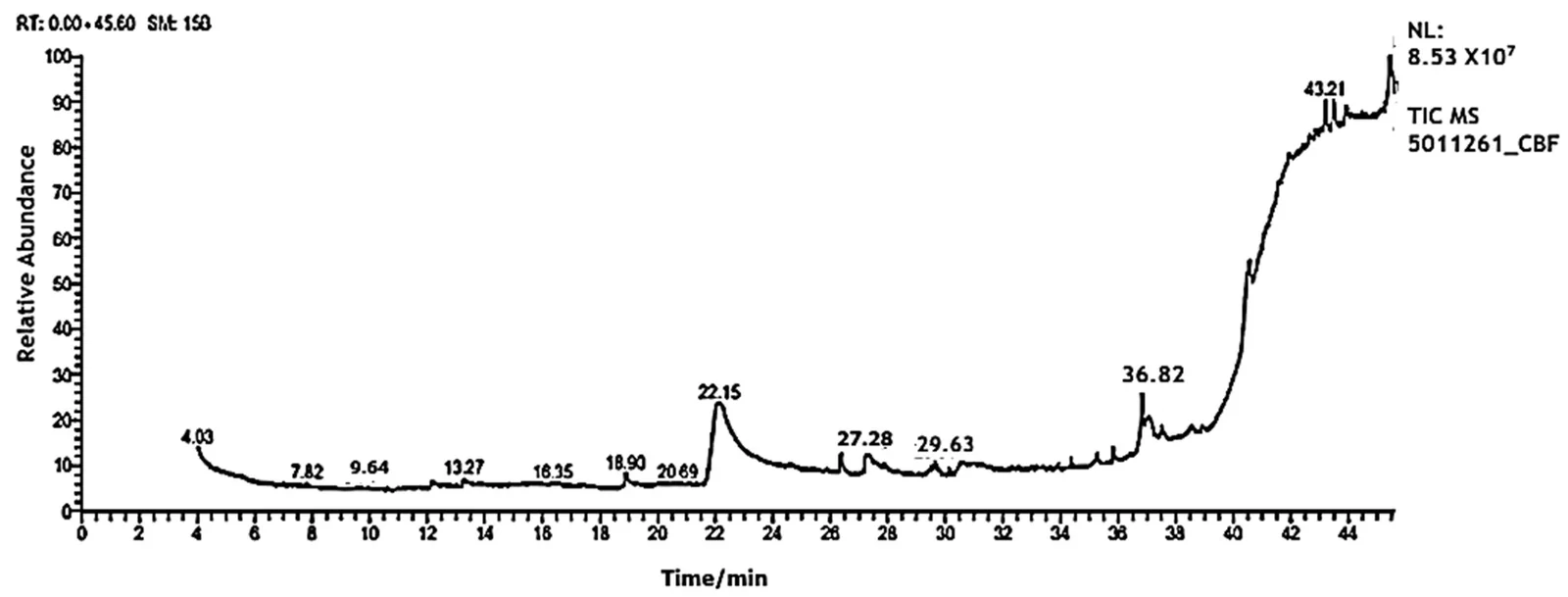
GC/MS Chromatograms of *C. edulis* flower methanolic extract.

To reveal the chemical architecture behind these biological phenotypes, the methanolic flower extract was subjected to Gas Chromatography–Mass Spectrometry (GC–MS) analysis. Structural annotations were validated by matching retention indices and fragmentation mass spectra against the National Institute of Standards and Technology (NIST) reference library. The analytical details—including systematic chemical identities, molecular formulas, retention times (Rt), and relative peak area percentages (%)—are combined in Table S1 in the associated supplementary file. The corresponding total ion chromatogram (TIC) is illustrated in Figure 6, showing a rich chemical variety dominated by bioactive triterpenoids, phytosterols (predominantly lupeol and *beta*-sitosterol), fatty acid derivatives, and highly substituted cyclitols. These metabolites make up the chemical matrix of the extract; which of them, if any, contribute to its activity was not tested. A stringent MF ≥ 800–850 threshold is generally recommended for confident GC-MS/NIST compound identification; however, complex crude plant extracts such as this one commonly contain co-eluting and structurally related compounds that depress match factors relative to analyses of pure standards. Consistent with common practice in phytochemical profiling of crude botanical mixtures, we therefore treated MF ≥ 650 as indicative of a tentative identification, while reserving the MF ≥ 800–850 range for confident identification. Compounds annotated below this confidence threshold (flagged in Table S1 in the data provided in supporting information) should accordingly be regarded as tentative pending orthogonal confirmation and were not used as the sole basis for the mechanism and in silico analyses that follow.

### 2.8. Molecular Docking Study

**Figure 7:**
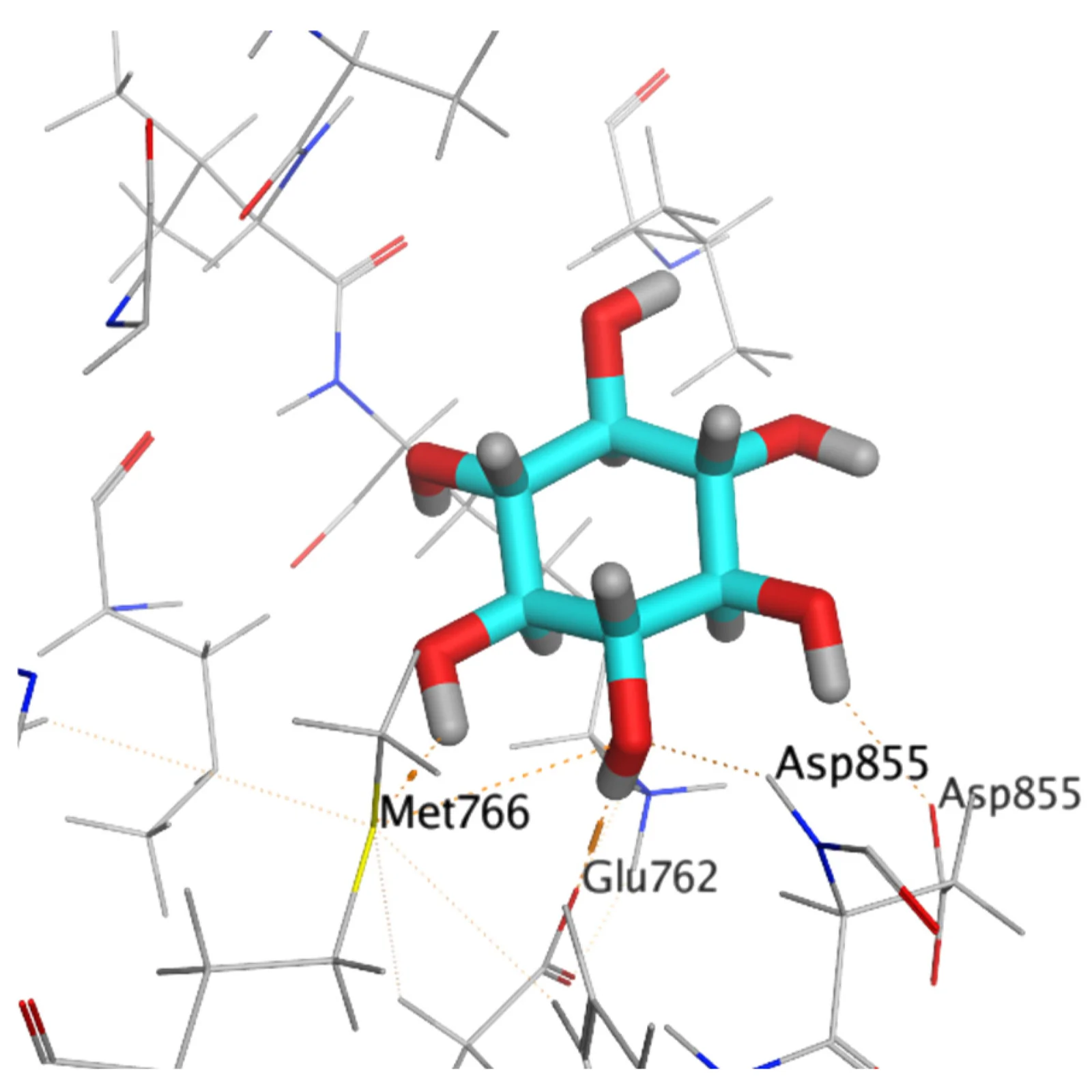
3D representation of compound mome inositol against EGFR.

To unravel the binding mechanics and identify the precise floral constituents driving the in vitro enzymatic inhibition of EGFR, structure-based molecular docking was executed using Molecular Operating Environment (MOE) software (version 2019.0102) against the high-resolution crystal structure of the human EGFR kinase domain (PDB ID: 3W33). The docking results revealed compound (mome inositol) as the top-ranked among the extract’s constituents, exhibiting the most favorable predicted docking score (−7.35 kcal/mol). This compound established five hydrogen bonds with Asp855, Glu762 and Met766 (Table 5, Figure 7). Compounds Cyclopropane butanoic acid, Hexadecanoic acid, 1,2,3-Propanetriol, 1,2-diacetate, beta-Sitosterol and Stigmast-5-en-3-ol, (3a,24S)-display moderate binding affinities. The docking protocol was validated by redocking the co-crystallized ligand into the EGFR active site, where it reproduced the native crystallographic pose with an RMSD of 1.71 Å, confirming the reliability of the adopted protocol. The redocked reference ligand obtained a docking score of −9.01 and formed two hydrogen bonds with Met766, together with a hydrophobic interaction with Phe856 (Table 5).

### 2.9. Molecular Dynamics Simulation

#### 2.9.1. Conformational Stability via Backbone RMSD

**Figure 8:**
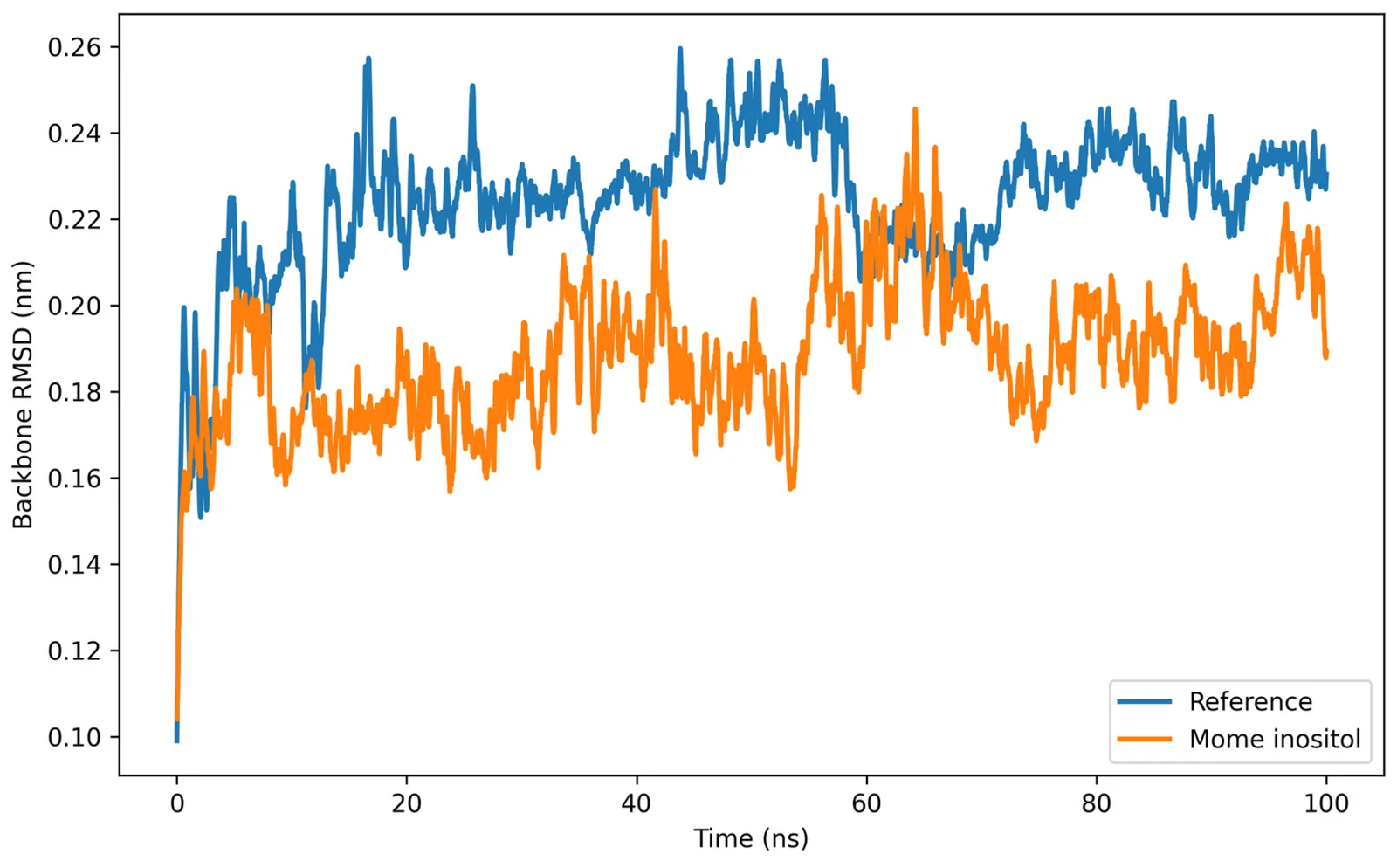
RMSD of both mome inositol–protein complex (orange) and reference co-crystallized ligand–protein complex (blue).

To cross-examine the structural validity of the docking pose and monitor the real-time thermodynamic behavior of the ligand within the catalytic site, a rigorous 100 ns molecular dynamics (MD) simulation was carried out. The structural stability of the protein–ligand complexes was evaluated by monitoring the backbone RMSD over a 100 ns molecular dynamics simulation (Figure 8). Both the mome inositol complex and the reference ligand complex rapidly attained equilibrium during the initial stage of the simulation and maintained stable trajectories thereafter. The mome inositol-bound system exhibited a lower average RMSD (0.188 nm) than the reference complex (0.224 nm), indicating enhanced structural stability. After the initial equilibration phase, both systems displayed only minor fluctuations without noticeable structural deviations, suggesting that neither complex underwent significant conformational changes throughout the simulation. The consistently reduced RMSD values observed for the mome inositol complex indicate that ligand binding effectively stabilized the protein backbone. Overall, the stable trajectories of both complexes, together with the lower RMSD values of the mome inositol system, predicted a favorable binding mode of mome inositol within the EGFR catalytic pocket. This supports the favorable binding mode predicted by the molecular docking study.

#### 2.9.2. Residue Flexibility Mapping via RMSF

**Figure 9:**
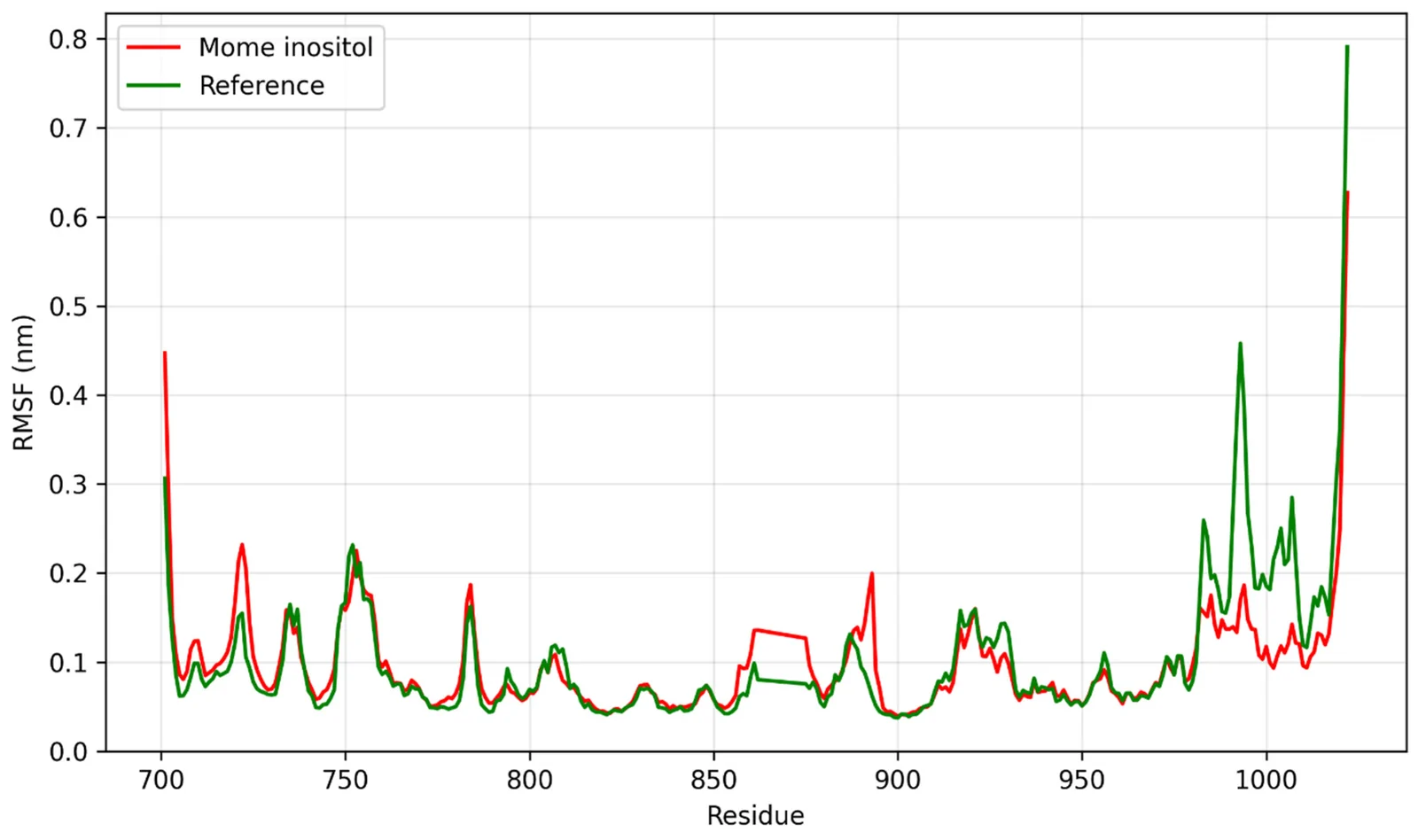
RMSF of both compound mome inositol–protein complex (red) and reference co-crystallized ligand–protein complex (green).

To characterize residue-level flexibility during the simulation, the root mean square fluctuation (RMSF) of each protein residue was calculated (Figure 9). The mome inositol and reference complexes displayed broadly similar fluctuation profiles throughout the 100 ns trajectory, indicating comparable overall conformational dynamics. Most residues within the simulated protein region (residues 701–1022) exhibited RMSF values below 0.15 nm, with higher fluctuations mainly observed in terminal and solvent-exposed loop regions. Quantitatively, the mome inositol complex showed a slightly lower average RMSF than the reference complex (0.096 vs. 0.102 nm) and a lower maximum fluctuation (0.627 vs. 0.791 nm; approximately 21% reduction), particularly toward the C-terminal region. These observations indicate that the mome inositol complex maintained a comparable residue-level dynamic profile to the reference complex, with modestly reduced fluctuations in some flexible regions. Collectively, the RMSF analysis provides computational support for the structural stability of the mome inositol–EGFR complex during the simulated trajectory, while the observed differences should not be interpreted as experimental evidence of EGFR inhibition or a definitive mechanism of action.

#### 2.9.3. Hydrogen Bond Persistence Analysis

**Figure 10:**
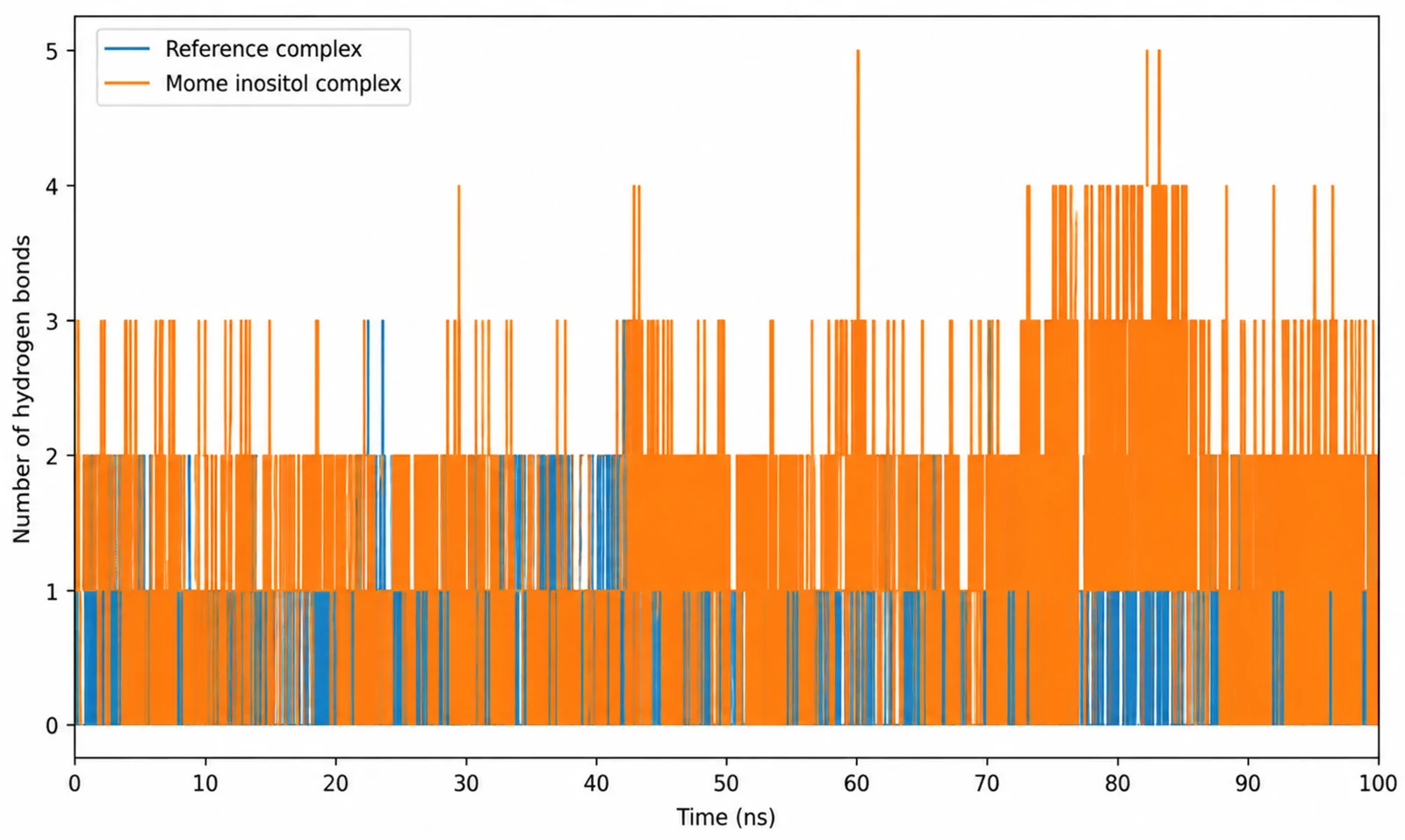
H-bonds of both compound mome inositol with target protein (orange) and reference co-crystallized ligand with target protein (blue).

The protein–ligand hydrogen-bond interactions were analyzed throughout the 100 ns molecular dynamics trajectory to assess the dynamic polar contacts within the binding interface (Figure 10). Both complexes exhibited fluctuations in hydrogen-bond formation throughout the simulation, reflecting the dynamic nature of protein–ligand interactions. The mome inositol complex generally maintained a higher number and greater frequency of hydrogen-bond interactions than the reference complex, with predominantly one to two hydrogen bonds and several transient increases to three or more hydrogen bonds, including occasional peaks of up to five hydrogen bonds. In contrast, the reference complex generally displayed fewer hydrogen-bond interactions, with most of the trajectory characterized by zero to one hydrogen bond and occasional transient increases. The more frequent hydrogen-bond formation observed for the mome inositol complex suggests persistent polar contacts with the EGFR binding site throughout the simulated period. Together with the RMSD and RMSF analyses, the hydrogen-bond profile provides additional computational support for the persistence and dynamic stability of the predicted mome inositol–EGFR interactions. However, these observations should be considered supportive computational evidence rather than definitive confirmation of binding affinity or inhibitory activity.

#### 2.9.4. Structural Compactness via Radius of Gyration

**Figure 11:**
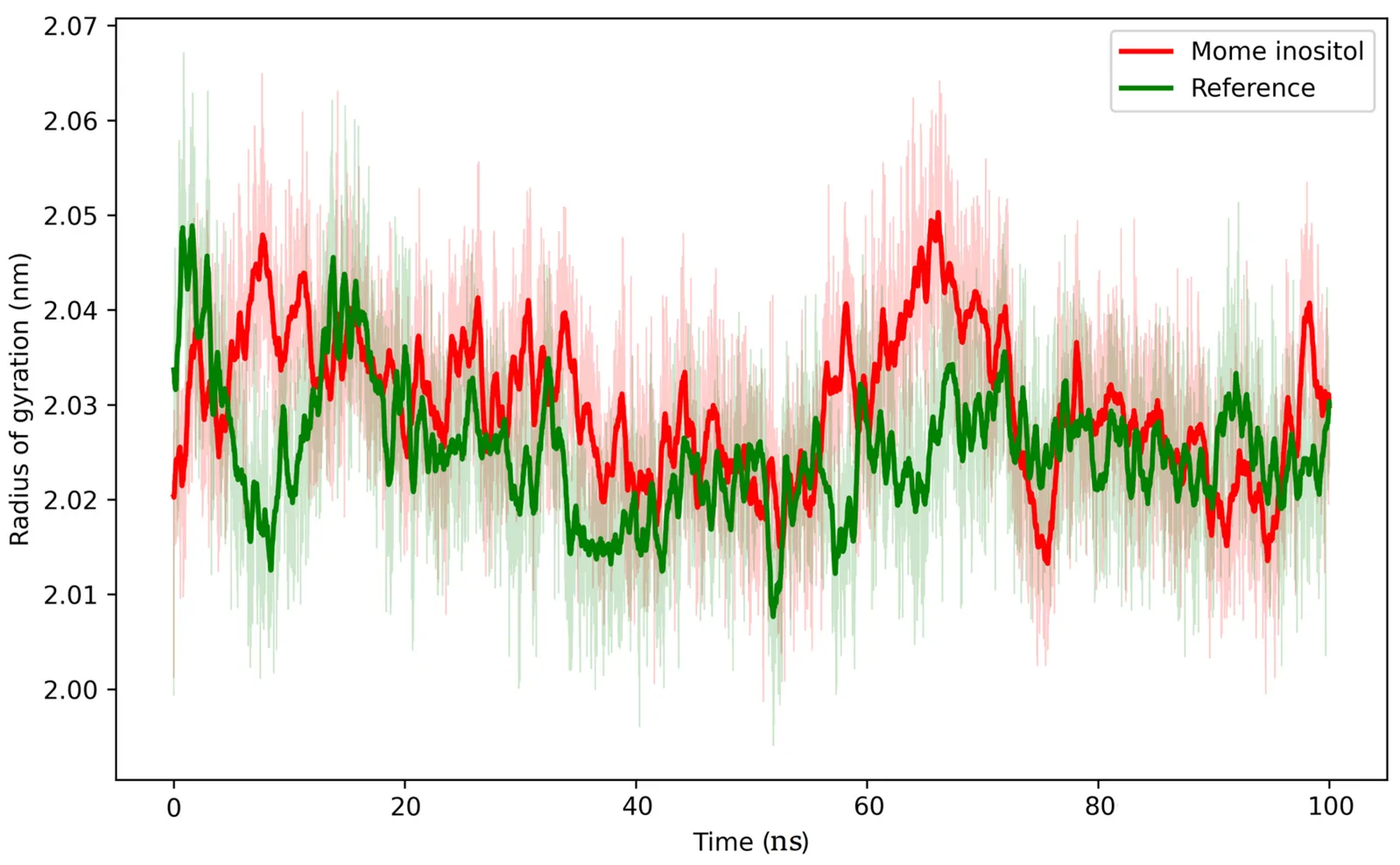
Radius of gyration (Rg) of both compound mome inositol–protein complex (red) and reference co-crystallized ligand–protein complex (green).

To assess potential changes in the global compactness and overall architecture of the protein upon ligand binding, the radius of gyration (Rg) was monitored throughout the 100 ns MD simulation (Figure 11). Both complexes exhibited relatively stable Rg profiles over the simulation period, indicating that their overall structural compactness was maintained. The mome inositol complex showed a slightly higher mean Rg compared with the reference complex, suggesting a marginal difference in global compactness without evidence of substantial structural expansion or collapse. The average Rg values were 2.030 ± 0.010 nm for the mome inositol complex and 2.025 ± 0.009 nm for the reference complex, indicating nearly identical structural compactness. No systematic increase or decrease in Rg was detected, suggesting that neither complex experienced unfolding or major conformational rearrangements. The stable Rg profiles indicate that ligand binding did not compromise protein folding. When interpreted alongside the RMSD, RMSF, and hydrogen-bond analyses, these results demonstrate that the mome inositol complex preserves a compact and dynamically stable conformation throughout the simulation, further supporting its favorable binding characteristics and potential as a promising lead compound.

## 3. Discussion

The clinical management of non-small cell lung cancer (NSCLC) remains severely hindered by the systemic toxicities and emergent mutational resistance associated with conventional therapies, driving the urgent exploration of multi-targeted, naturally derived oncological agents. The findings of this study highlight the antiproliferative activity of the methanolic flower extract of *Carpobrotus edulis* against the investigated cancer cell lines, with A-549 human lung cancer cells showing the highest susceptibility (IC_50_ = 3.62 ± 0.34 µg/mL). However, selectivity toward cancer cells relative to normal cells was not assessed in the present study. Flow cytometry and evaluation of apoptosis-related biomolecules collectively revealed that this cytotoxic effect of the extract was associated with a significant accumulation of cells in the S phase of the cell cycle and activation of apoptotic pathways. This effect was accompanied by the coordinated modulation of p53, Bax, Bcl-2, and caspase-3 detailed in Section 2.5 (Table 4), supporting the involvement of mitochondrial-mediated apoptosis in the observed antiproliferative activity. Collectively, these findings indicate that the extract promotes apoptotic cell death rather than passive, inflammation-associated necrosis [12,14,15,18].

The *C. edulis* flower methanolic extract demonstrated notable antioxidant activity in both ABTS and H_2_O_2_ scavenging assays, comparable to the reference antioxidant. Similar observations have been reported for other medicinal plant extracts, where antioxidant potential was found to coexist with antiproliferative activity. *Sisymbrium officinale* extracts exhibited significant antioxidant activity alongside inhibitory effects against cancer cell lines, while mixtures of Thai medicinal plants similarly demonstrated concurrent antioxidant and anticancer properties [19,20]. Collectively, these studies support the view that plant extracts rich in bioactive phytochemicals may possess multiple biological activities, including antioxidant and antiproliferative effects. However, the present study did not directly investigate whether the observed antiproliferative activity of *C. edulis* was mediated through antioxidant mechanisms; therefore, a direct mechanistic relationship between these activities cannot be established and warrants further investigation.

The GC-MS profile of the extract contains cyclitols, phytosterols, fatty acids and triterpenoids; whether they act together or separately was not tested. The top-ranked metabolite in the in silico screen was the cyclitol derivative mome inositol.

Mome inositol is an inositol-related sugar alcohol that occurs naturally in plants and microorganisms and is essential for multiple biological functions [21], and has previously been reported at high concentration in the stems of *Mesembryanthemum crystallinum* L., another Aizoaceae species [22]; a related inositol derivative was likewise identified in *C. edulis* stem extract [23]. Several of the other major metabolites identified here have comparable precedent within the family: hexadecanoic acid derivatives have been reported in *C. edulis* leaf and stem essential oils [23,24] and in *Sesuvium portulacastrum* [25] and *Opophytum forskahlii* seed oil [26]. The propanetriol derivative, galactosyl diacylglycerol, was previously isolated from *C. edulis* methanolic extract [27,28]. The compound cyclopropane butanoic acid has been reported in *Opophytum forskahlii* seed oil [26]. Sitosterol compounds are documented across the family in *Trianthema portulacastrum* [25] and *Mesembryanthemum nodiflorum* [29].

The anticancer literature summarized below pertains specifically to the well-characterized D-pinitol stereoisomer; its relevance to our extract’s metabolite is contingent on the orthogonal stereochemical confirmation discussed above.

Several of these compounds have documented anticancer activities. For example, in rat breast carcinogenesis produced by 7,12-dimethylbenz(a)anthracene (DMBA), D-pinitol decreased breast cancer and induced apoptosis. In addition to reducing tumor volume, D-pinitol therapy also inhibited nuclear factor kappa B (NF-κB) activation in rat tumors, which in turn modulates its downstream p53 and caspase-3 family proteins [30]. Hexadecanoic acid (palmitic acid) has been shown to be effective against a number of cancers, including colorectal, gastric, liver, cervical, and breast cancers [31]. Its anti-tumor activities include causing tumor cells to undergo apoptosis, preventing tumor cell growth, preventing invasion and metastasis, increasing chemotherapy sensitivity, and strengthening the immune system [31]. Palmitic acid’s primary anticancer mechanism is the stimulation of cell death via the mitochondrial route, which is made possible by the enhancement of intracellular production of reactive oxygen species. Additionally, palmitic acid interferes with the cancer cell cycle, causing cell cycle arrest primarily in the G1 phase [31]. Palmitic acid slowed cell migration, invasion, and angiogenesis, triggers programmed cell autophagy death and worked in concert to increase the effectiveness of chemotherapeutic medications while lowering side effects [31].

Many researchers have reported that β-sitosterol effectively obstructs cancer cell proliferation. Several studies have demonstrated the remarkable ability of β-sitosterol and its derivatives to induce apoptosis in various cell lines by promoting apoptosis through modulation of FAS levels and Caspase-8 activity, phosphorylation of extracellular signal-regulated kinase (ERK), and p38 mitogen-activated protein kinase [32,33,34,35]. Additionally, it controls the activity of antioxidant enzymes during pathogenesis, decreases the generation of free radicals, and induces G2/M phase cell cycle arrest. Numerous therapeutic benefits of lupeol have been documented, including potent antioxidant, antimutagenic, anti-inflammatory, and anti-arthritic activities. Lupeol’s ability to alter signaling pathways like nuclear factor kappa B (NFjB) and the phosphatidylinositol 3-kinase (PI3 K)/Akt (protein kinase B pathway), which are said to be crucial during tumorigenesis, has been linked to its anti-tumor promotion effects [33]. The activity of ornithine decarboxylase (ODC) protein, a well-known indicator of tumor promotion, was found to be considerably inhibited by lupeol [36]. Lupeol causes human promyelocytic HL-60 leukemia cells to undergo apoptosis, according to [37]. When exposed to lupeol for 72 h, cancer cell lines with various histological origins—such as T-lymphoblastic leukemia CEM, breast carcinoma MCF-7, lung carcinoma A-549, multiple myeloma RPMI 8226, cervical carcinoma HeLa, and malignant melanoma G361—die [38]. Additionally, lupeol has been shown to prevent the ERα-negative breast cancer cells MDA-MB231 from proliferating [39].

These findings support the hypothesis that the anticancer activity of *C. edulis* is mediated by a combination of phytosterols, terpenoids, and fatty acids. A limitation of the present study is the absence of a normal cell line for comparison. Consequently, the results demonstrate antiproliferative activity against the cancer cell lines investigated but do not allow conclusions regarding selective toxicity toward malignant cells. Future studies incorporating appropriate normal human cell lines will be necessary to assess the therapeutic selectivity and safety profile of the extract.

EGFR is a clinically validated molecular target in NSCLC and plays an important role in lung cancer-associated signaling, including pathways regulating cell proliferation and survival [40,41]. EGFR expression and functional signaling have been demonstrated in A549 cells, supporting its biological relevance to the cellular model used in the present study [41,42]. The molecular docking and molecular dynamics simulation results provide computational support for a favorable and dynamically stable interaction of mome inositol with EGFR. Molecular docking identified mome inositol as the top-ranked metabolite, exhibiting the highest binding affinity (−7.35 kcal/mol) among the screened phytochemicals. The ligand established multiple hydrogen bonds with key active-site residues of the binding pocket that were defined by the co-crystallized ligand, including Asp855, Glu762, and Met766, together with favorable hydrophobic interactions. Interestingly, these findings are supported by a recent computational study by Shukla et al. [43] that investigated 1-DL-(indole-3-acetyl)-mome inositol (M1) as a potential EGFR inhibitor. Analysis of the reported 2D docking interaction profile of M1 showed successful hydrogen-bonding interactions between its inositol moiety and EGFR residues Asp855 and Glu762, similar to the interaction pattern observed for the inositol moiety of mome inositol in the present study. This recurrence of key EGFR residues across independent computational investigations further supports the potential importance of the inositol scaffold in mediating favorable interactions within the EGFR kinase domain. The docking study is aligned with previous biological research suggesting that inositol derivatives may influence the EGF/EGFR-related signaling pathway of epidermal growth factors (EGF) [44,45], which is consistent with these observations. The present study demonstrated inhibitory activity of both the flower extract and mome inositol against the EGFR kinase enzyme in a cell-free assay. This agreement is limited to the cell-free kinase assay and does not show inhibition of EGFR in cells. Although EGFR expression and signaling have been reported in A549 cells, the presence of the activating KRAS G12S mutation may partially uncouple downstream signaling from EGFR activity, thereby limiting direct mechanistic interpretation of the observed cellular responses.

The docking predictions were further investigated through a 100 ns molecular dynamics (MD) simulation to evaluate the temporal stability and conformational behavior of the predicted mome inositol–EGFR complex. Molecular docking provides a structural prediction of a possible ligand-binding mode, whereas MD simulations allow assessment of the dynamic behavior and stability of the predicted protein–ligand complex [46,47]. In the present study, complementary trajectory-based parameters, including backbone RMSD, residue-level RMSF, hydrogen-bond persistence, and radius of gyration, were analyzed to characterize the stability, flexibility, intermolecular contacts, and overall compactness of the simulated complexes [48]. The mome inositol complex exhibited lower RMSD and RMSF values, indicating enhanced structural stability and reduced residue mobility. Furthermore, the ligand maintained a greater number of persistent hydrogen bonds while preserving the compactness of the protein, as demonstrated by the stable radius of gyration. Collectively, the RMSD, RMSF, hydrogen-bond persistence, and radius-of-gyration analyses provide complementary computational evidence supporting the conformational stability and persistence of the predicted mome inositol–EGFR complex during the 100 ns simulation. Nevertheless, these computational observations should be distinguished from direct experimental determination of ligand–EGFR binding affinity. Molecular docking and molecular dynamics describe a predicted interaction and its behavior under the simulated conditions, but do not independently establish direct physical target engagement [49]. Accordingly, the present findings support EGFR as a putative molecular target of mome inositol and provide a testable structural hypothesis that warrants further experimental validation.

The findings presented in this work are supported exclusively by cellular in vitro assays and in silico simulations. No in vivo anti-tumor efficacy evaluation in a tumor-bearing animal model has yet been performed for either the crude extract or mome inositol; accordingly, their tumor-suppressive activity, pharmacokinetic behavior, and overall in vivo druggability remain to be established. Pharmacokinetic behavior of the extract’s bioactive constituents has likewise not been characterized. The absorption, distribution, metabolism, and excretion (ADME) profiles of mome inositol and the other major metabolites—and consequently their bioavailability and feasibility for a defined route of administration—remain undetermined. Similarly, no systematic structure–activity relationship (SAR) investigation was undertaken. The present work identifies mome inositol’s favorable in silico interaction with EGFR but does not extend to structural modification or comparative activity testing of related cyclitol or phytosterol analogs; therefore, it does not yet provide guidance for lead compound optimization. Building on the planned in vivo xenograft study, future work will include pharmacokinetic characterization of the extract’s principal metabolites and the synthesis of a focused panel of mome inositol and phytosterol structural analogs for comparative EGFR-binding and cytotoxicity screening. Together, these efforts aim to establish a preliminary SAR profile to guide lead optimization and support the translational development of this natural anticancer candidate.

### Limitations of This Study

The main limitations of this study are as follows. (i) No non-malignant cell line was tested, so selectivity cannot be assessed. (ii) The MTT assay measures metabolic activity and not cell number. (iii) A-549 cells carry a KRAS G12S mutation, EGFR was tested only on the purified enzyme, and phosphorylated EGFR and downstream signaling were not examined in cells. (iv) The caspase-3 ELISA did not distinguish pro-caspase-3 from the cleaved form, and mitochondrial markers other than Bax and Bcl-2 were not measured. (v) Antioxidant activity was measured only in cell-free assays. (vi) Docking scores are approximate and were not converted to binding free energies, and the identity of mome inositol rests on a library match.

## 4. Materials and Methods

### 4.1. Chemicals and Reagents

All solvents and mome inositol powder used in this study were of analytical grade and purchased from Sigma Aldrich, St’ Louis, MO, USA. Fisher Scientific, Waltham, MA, USA. Scharlau Sentmenat, Barcelona, Spain, and VWR BDH Prolabo Chemical, Fontenay-sous-Bois, France. Roswell Park Memorial Institute Medium (RPMI-1640), 4-(2-hydroxyethyl)-1-piperazineethanesulfonic acid (HEPES), L-glutamine, and 0.25% Trypsin-EDTA were obtained from Lonza, Verviers, Belgium.

### 4.2. Plant Material and Extraction

*Carpobrotus edulis* fresh flowers were collected from Al-Namas, Asir region of Saudi Arabia, where the plant is considered invasive (19°09′23.1″ N 42°06′46.0″ E) in spring (20 April 2023). The taxonomic identity of the plant was confirmed by Dr. Hamdoon Mohammed, Qassim University, who identified the plant and verified the name using https://www.worldfloraonline.org/taxon/wfo-0001435059 in 30 April 2023,and a voucher specimen was kept in the Department of Pharmaceutical Chemistry, University of Ha’il, Hail, Saudi Arabia as (UOHCOP009). The collected plant material (flowers) was air-dried, powdered and macerated several times with 70% methanol till exhaustion. The collected extracts were filtered and concentrated under reduced pressure to obtain the crude methanolic flower extract.

### 4.3. Cytotoxic Activity

#### 4.3.1. Cell Lines

The antiproliferative activity of the flower methanolic extract was evaluated against four human cancer cell lines: A-549 (lung adenocarcinoma), Panc-1 (pancreatic carcinoma), HepG2 (hepatocellular carcinoma), and MCF-7 (breast adenocarcinoma). All cell lines were obtained from the American Type Culture Collection (ATCC, Rockville, MD, USA).

#### 4.3.2. Cell Line Propagation

A-549 and HepG2 cells were cultured in Dulbecco’s Modified Eagle Medium (DMEM) supplemented with 10% heat-inactivated fetal bovine serum (FBS). Panc-1 and MCF-7 cells were maintained in RPMI-1640 medium supplemented with 10% FBS. All cultures were incubated at 37 °C in a humidified atmosphere containing 5% CO_2_ and sub-cultured 1–2 times weekly [50,51].

#### 4.3.3. MTT Cytotoxicity Assay

Cytotoxicity was assessed using the MTT assay. Cells were seeded in 96-well plates at a density of 5 × 10^4^ cells/well and incubated for 24 h. Serial dilutions of the extract (0.25–500 µg/mL) were added and incubated for 72 h. After treatment, the medium was replaced with 100 µL of fresh medium containing 10 µL of 12 mM MTT and incubated for 4 h. Subsequently, 85 µL of the medium was removed, and 50 µL of DMSO was added to dissolve the formazan crystals. Absorbance was measured at 590 nm using a microplate reader (SunRise, Tecan US, Inc., Morrisville, NC, USA). The assay reports metabolic activity, which is used here as a measure of viability.

Cell viability (%) was calculated as:

$$\mathrm{Viability}=\frac{{\mathrm{OD}}_{t}}{{\mathrm{OD}}_{c}}\times 100$$

where (*OD_t_*) and (*OD_c_*) represent treated and control absorbance values, respectively. IC_50_ values were determined from dose–response curves. Vinblastine sulfate served as the positive control for the cytotoxicity assays [52,53].

### 4.4. Antioxidant Activity

The antioxidant potential of the flower methanolic extract was evaluated using the ABTS radical scavenging assay and the hydrogen peroxide (H_2_O_2_) scavenging assay following previously described protocols [52,53]. Ascorbic acid served as the standard antioxidant.

### 4.5. Cell Cycle Analysis

Cell cycle distribution in A-549 cells was assessed using the CycleTEST™ PLUS DNA Reagent Kit (BD Pharmingen™, Heidelberg, Germany). A-549 cells were seeded and allowed to attach overnight before treatment with the IC_50_ (3.62 µg/mL) of the flower methanolic extract for 72 h. Untreated cells served as the negative control. Following treatment, cells were harvested, washed with phosphate-buffered saline (PBS), and processed according to the manufacturer’s instructions. Cellular DNA was stained with propidium iodide, and DNA content was analyzed using a BD FACS Calibur flow cytometer (BD Biosciences, San Jose, CA, USA). Cell-cycle distribution was determined as the percentage of cells in the G0/G1, S, and G2/M phases using CellQuest software, Version 7.5.3. The experiment was performed in three independent biological replicates, and data are presented as mean ± SD.

#### 4.5.1. Apoptosis Analysis (Annexin V-FITC Assay)

Apoptosis was evaluated using the Annexin V-FITC assay/propidium iodide (PI) staining [54]. Briefly, A549 human lung adenocarcinoma cells were treated with the IC_50_ (3.62 µg/mL) of the flower methanolic extract for 72 h, harvested, washed twice with PBS, and resuspended in binding buffer. A 100 μL aliquot of the cell suspension was incubated with 1 μL of FITC-conjugated Annexin V (BD Pharmingen™, Heidelberg, Germany) for 15-20 min at room temperature in the dark. Subsequently, propidium iodide (PI) was added according to the manufacturer’s instructions. Samples were immediately analyzed using a BD FACS Calibur flow cytometer (BD Biosciences, San Jose, CA, USA). Data were analyzed to determine the percentages of viable, early apoptotic, late apoptotic, and necrotic cells.

#### 4.5.2. ELISA Analysis of Apoptotic Markers

A-549 cell lysates (treated and untreated) were analyzed for Bax, Bcl-2, p53, and caspase-3 using commercial ELISA kits: Bax (MBS935667; sensitivity 15.6 pg/mL), Bcl-2 (BMS244-3; sensitivity 0.5 ng/mL), p53 (MBS355295; sensitivity 10 pg/mL), and Caspase-3 (MBS261814; sensitivity 0.5 ng/mL). All kits exhibited intra- and inter-assay CV < 15%. Absorbance was measured at 450 nm using a Hidex Sense ELISA Multimode Reader (Hidex Oy, Turku, Finland).

### 4.6. EGFR Kinase Inhibition Assay

EGFR activity was evaluated using a commercial EGFR Assay Kit (BPS Bioscience, San Diego, CA, USA, Catalog # 40321). The assay employs recombinant human EGFR kinase and the Poly(Glu:Tyr 4:1) substrate, with kinase activity quantified indirectly through ATP consumption using the Kinase-Glo^®^ MAX luminescent detection system.

Briefly, reaction mixtures containing kinase buffer, ATP, Poly(Glu:Tyr 4:1) substrate, recombinant human EGFR enzyme, and the test sample were prepared in 96-well microplates according to the manufacturer’s instructions. The flower extract, mome inositol, and sorafenib were prepared over a concentration range of 0.25–500 µg/mL. Vehicle-treated reactions containing ≤1% (*v*/*v*) DMSO served as the negative control. Following incubation at 30 °C for the time specified by the manufacturer, Kinase-Glo^®^ MAX reagent was added, and luminescence was measured using a Hidex Sense multimode microplate reader [55]. EGFR kinase inhibition (%) was calculated relative to the vehicle-treated control, and concentration-response curves were generated to determine inhibitory activity. All experiments were performed in triplicate, and results are presented as mean ± SD.

### 4.7. GC–MS Analysis

Chemical profiling of the flower methanolic extract was performed using a Thermo MS-DSQ II system coupled to a TRACE Ultra GC (Thermo Scientific, Waltham, MA, USA). Separation was achieved on a DB-5 capillary column (30 m × 0.25 mm × 0.25 µm). Injection volume was 1 µL, helium was used as the carrier gas (1.0 mL/min), and the oven temperature was programmed from 70 °C to 260 °C at 6 °C/min. The injector and MS transfer line temperatures were 270 °C and 260 °C, respectively. Compounds were identified by comparing mass spectra with the NIST library.

### 4.8. In Silico Docking and Molecular Dynamics

#### 4.8.1. Molecular Docking

Molecular docking simulations were performed for the major metabolites identified in the methanolic flower extract of *C. edulis*, selected based on their relative GC–MS peak abundances (Table S1), using the Molecular Operating Environment (MOE) software (version 2019.0102) [56,57]. The metabolites were sketched, protonated, assigned partial charges, energy-minimized, and saved as an MOE database (.mdb) [58,59]. The crystal structure of the EGFR kinase domain (PDB ID: 3W33) was retrieved from the Protein Data Bank and prepared using the MOE QuickPrep protocol by removing water molecules, adding hydrogen atoms, and protonating the protein. Docking was carried out within the ATP-binding pocket defined by the co-crystallized ligand using the Triangle Matcher placement algorithm, with London dG as the initial scoring function, force field refinement, and GBVI/WSA dG for final rescoring under the Amber10 force field [60,61]. Protein–ligand interactions were evaluated based on docking scores and binding poses. The docking protocol was validated by redocking the co-crystallized ligand (compound 19b) into the EGFR active site, yielding an RMSD of 1.71 Å relative to its native crystallographic pose, thereby confirming the reliability of the adopted docking protocol.

#### 4.8.2. Molecular Dynamics Simulation

All-atom molecular dynamics simulation was performed for the selected protein–ligand complexes using GROMACS 2020.3. The calculations were done on a GPU-enabled system using an NVIDIA Quadro T2000 GPU. AMBER99SB all-atom force field was used to generate protein topology, while ligands were parameterized using the acpype server. Complexes were built using ligand coordination obtained from docking studies, and then complexes were boxed in a dodecahedron box and solvated with TIP3P explicit water molecules. Systems were neutralized by the addition of the required number of Na^+^ or Cl^−^ ions. The system’s energy was minimized using the steepest descent algorithm with a maximum force of 1000 kJ mol^−1^ nm^−1^. Equilibration using NVT and NPT ensembles for 100 ps each was done afterward, and then a production run was done for 100 ns. Temperature was controlled at 300 K using the V-rescale algorithm, while pressure was maintained using the Parrinello–Rahman barostat as required. The Linear Constraint Solver (LINCS) algorithm was used for bond-length constraints, and the Particle Mesh Ewald (PME) method was used for long-range electrostatics calculations. For all simulations, a 2 fs timestep was used. Van der Waals cut-off distance (rvdw) was set to 1.2 nm. Trajectories from the production run were used for analysis after correction of periodic boundary conditions (PBC) using trajconv [57].

### 4.9. Statistical Analysis

All experiments were performed in triplicate, and results are expressed as mean ± standard deviation (SD). IC_50_ values were calculated using nonlinear regression in GraphPad Prism 7 (GraphPad Software, San Diego, CA, USA). Statistical comparisons of the cytotoxic effects among the four cancer cell lines and the positive control were performed using one-way ANOVA followed by Tukey’s post hoc test. Comparisons between untreated and treated groups in the biological assays, including cell-cycle analysis, were performed using an independent-samples Student’s *t*-test. Differences were considered statistically significant at *p* < 0.05.

## 5. Conclusions

This preliminary in vitro and in silico study shows that *Carpobrotus edulis* methanolic flower extract reduced the viability of four human cancer cell lines, with the lowest IC_50_ in A-549 non-small cell lung cancer cells (3.62 ± 0.34 µg/mL). In A-549 cells the extract increased the S-phase fraction and the Annexin V-positive population and changed p53, Bax and Bcl-2 in a pattern consistent with the intrinsic apoptotic pathway. The extract also scavenged radicals in cell-free assays.

Through integrated GC-MS chemical profiling and advanced computational modeling, the cyclitol derivative mome inositol was identified as the top-ranked candidate scaffold from the in silico screen, pending confirmation of its identity and abundance. Mome inositol is a candidate for further study as an EGFR-directed compound, and these findings suggest that *C. edulis* flowers are a source of compounds that merit further investigation. Future research will focus on in vivo translational validation, formal pharmacokinetic profiling, and structure–activity relationship (SAR) optimizations to advance these natural candidate scaffolds toward clinical oncology applications.

## Figures and Tables

**Table 1:** IC_50_ (µg/mL) of *C. edulis* flower methanolic extract and vinblastine sulfate against HepG-2, A-549, Panc-1, and MCF-7 cancer cells.

Sample	IC_50_ (µg/mL)			
	HepG-2	A-549	Panc-1	MCF-7
Flower methanolic Extract	4.86 ± 0.48 ^b^	3.62 ± 0.34 ^b^	4.17 ± 0.32 ^b^	6.79 ± 0.45 ^b^
Vinblastine sulfate	0.89 ± 0.13	6.14 ± 0.43	1.67 ± 0.19	3.02 ± 0.26

Values are expressed as mean ± SD (n = 3). ^b^: Significant difference compared with positive control group.

**Table 2:** IC_50_ (µg/mL) of *C. edulis* flower methanolic extract using H_2_O_2_ and ABTS radical scavenging assays.

Sample	ABTS Radical Scavenging IC_50_ (µg/mL)	H_2_O_2_ Radical Scavenging IC_50_ (µg/mL)
Flower methanolic Extract	8.51 ± 0.79 ^b^	10.44 ± 2.19 ^b^
Ascorbic acid	13.65 ± 0.48	12.51 ± 0.56

Values are expressed as mean ± SD (n = 3). ^b^: Significant difference compared with control group.

**Table 3:** Effects of *C. edulis* flower methanolic extract on cell-cycle distribution in A-549 cells, as determined by flow cytometric analysis.

Sample Code	Tested Conc. (µg/mL)	Cell Population % ^a^		
		%G0–G1	%S	%G2-M
Flower methanolic Extract	3.62	45.26 ^b^ ± 1.10	45.42 ^b^ ± 1.4	9.31 ^b^ ± 0.91
Untreated A-549	0	50.72 ± 1.35	36.43 ± 4.8	12.84 ± 1.52
*p*-value		0.006	0.004	0.036

^a^ Cell-cycle distributions are expressed as mean ± SD from three independent experiments (n = 3). ^b^ Significance responses of *C. edulis* flower methanolic extract treatments vs. untreated A-549 cell lines.

**Table 4:** Effect of IC_50_ (µg/mL) *C. edulis* flower methanolic extract on Bax, Bcl-2, caspase-3, and p53 protein levels in A-549 cells.

Sample	Apoptotic Markers Concentration (ng/mL) ^a^			
	Bax	Bcl-2	Caspase-3	p53
Flower methanolic Extract	20.07 ± 0.61 ^b^	3.38 ± 0.51 ^b^	31.94 ± 11.38 ^b^	14.15 ± 0.42 ^b^
Untreated A-549	4.19 ± 0.42	7.79 ± 0.55	59.41 ± 3.92	3.91 ± 0.24

^a^ Concentration of apoptotic markers data is presented as mean ± SD from a triplicate experiment. ^b^ Significance responses of *C. edulis* flower methanolic extract treatments vs. untreated A-549 cell lines.

**Table 5:** Docking simulation results of eight major compounds of *C. edulis* methanolic flower extract against EGFR.

Compound Name	Docking Score kcal/mol	Key Residues	Binding Mode	2D Representation
Mome inositol	−7.35	Asp855 Glu762 Met766	2 H-Bond H-Bond 2 H-Bond	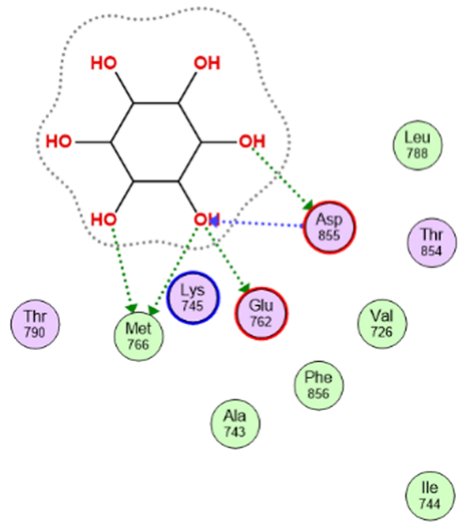
Cyclopropanebutanoic acid	−6.97	Lys716 Lys728	2 H-Bond Ionic interaction	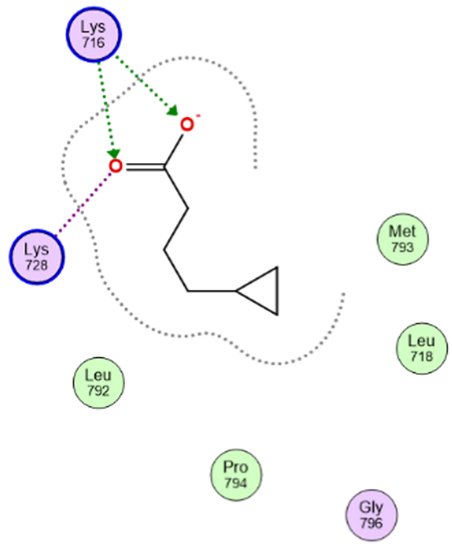
Hexadecanoic acid	−6.73	Lys716 Lys728 Lys728	H-Bond H-Bond Ionic interaction	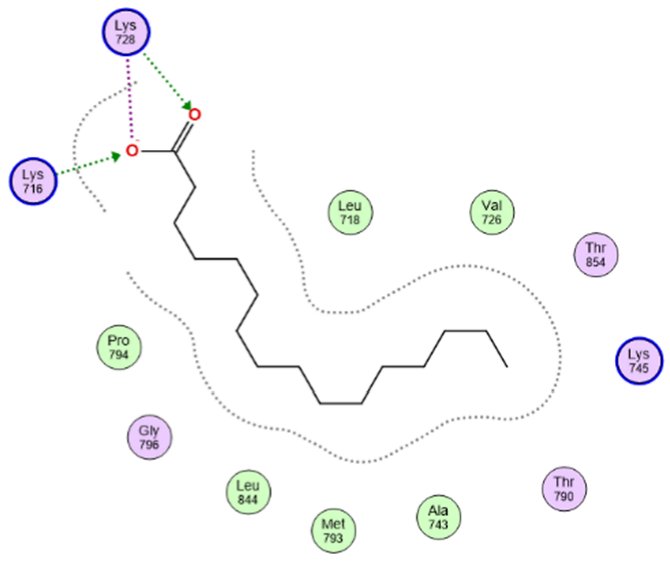
1,2,3-Propanetriol, 1,2-diacetate	−5.01	Lys716 Lys728	H-Bond H-Bond	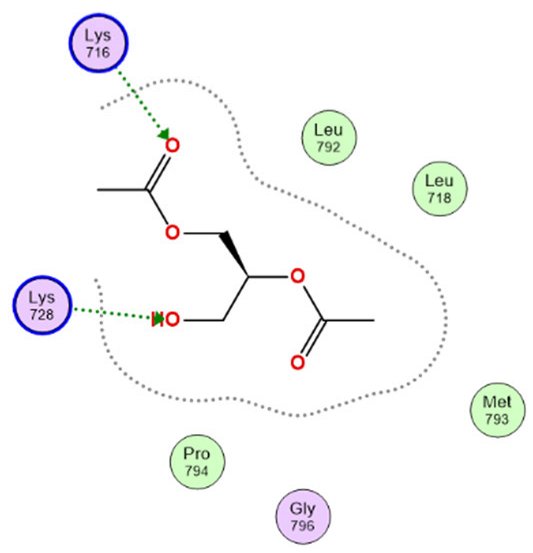
beta-Sitosterol	−4.97	Asp855 Met766	H-Bond H-Bond	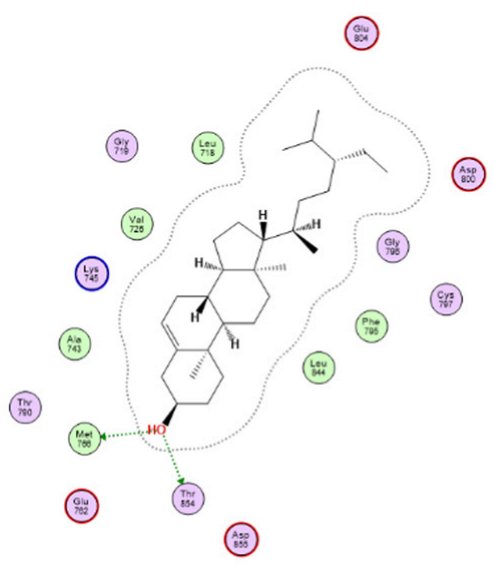
Stigmast-5-en-3-ol, (3a,24S)-	−4.92	Lys728	H-Bond	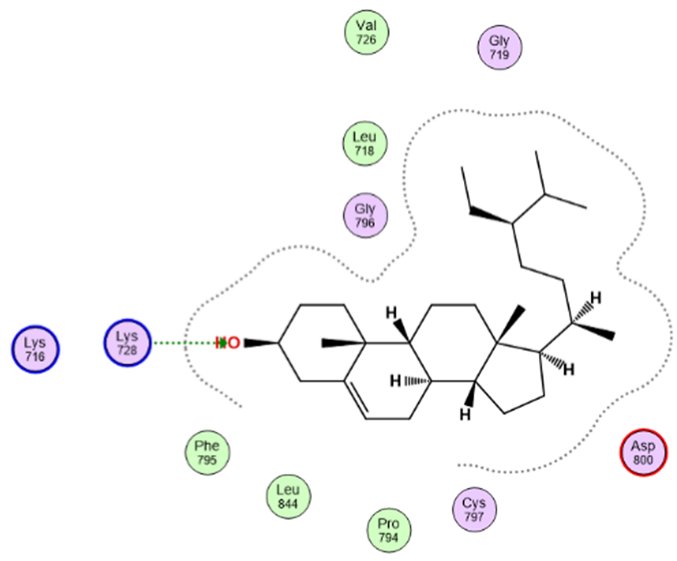
1H-cycloprop[e]azulen-7-ol	−4.91	Asp855	H-Bond	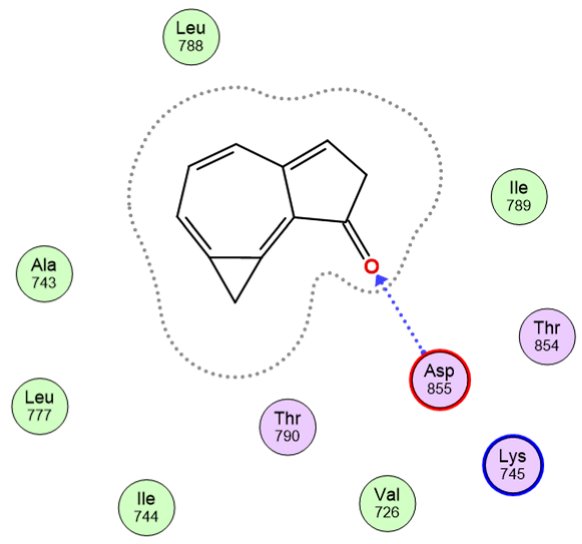
Lupeol	−4.80	Lys728	H-Bond	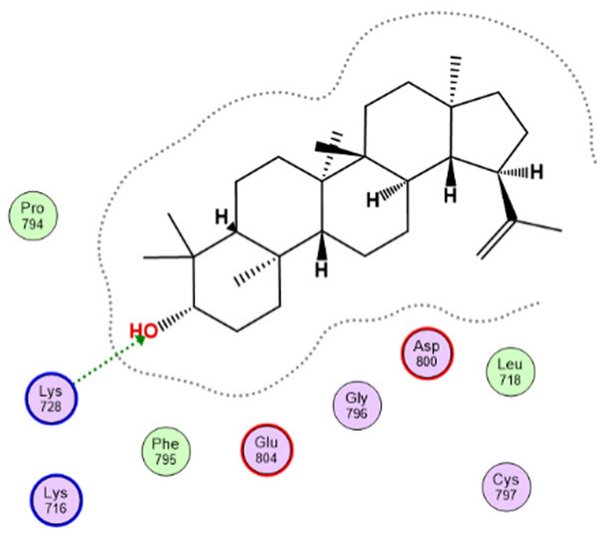
Co-crystal ligand	−9.0082	Met793Phe856	2 H-Bond Hydrophobic interaction	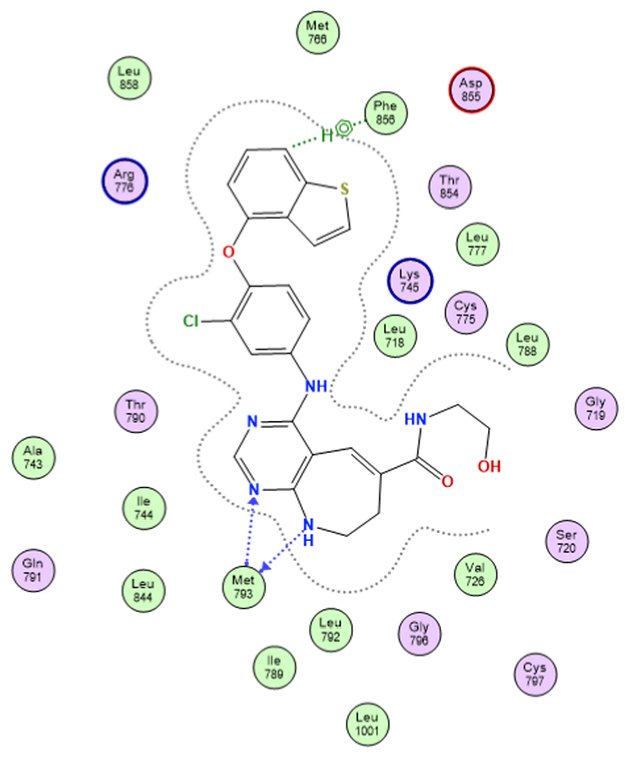

## Data Availability

The original contributions presented in this study are included in the article. Further inquiries can be directed to the corresponding authors.

## Supplementary Materials

The following supporting information can be downloaded at https://www.mdpi.com/article/10.3390/ijms272110016/s1.
