# Supplementary material for: Carpobrotus edulis Flower Extract Exerts Cytotoxic and Apoptotic Effects on A-549 Lung Cancer Cells: Phytochemical Profiling and In Silico EGFR Interaction Screening
Source: Int J Mol Sci. 2026 Nov 8;27(21):10016. doi: 10.3390/ijms272110016 (PMC13764303; doi:10.3390/ijms272110016)
Supplement: Supplementary file 1 [file ijms-27-10016-s001.zip › ijms-4670927-supplementary.pdf]

SUPPORTING INFORMATION

Carpobrotus edulis Flower Extract Exerts Cytotoxic and Apoptotic Effects on A-549 Lung Cancer Cells: Phytochemical Profiling and *In Silico* EGFR Interaction Screening

Bader Huwaimel [1,2], Amr S. Abouzied [1,2,*], Marwa M. Abd Rabo [3], Mohamed A. Said [4,5], Saad Alqarni [1,2], Ali Alghubayshi [2,6], Hashem O. Alsaab [7], Rami M. Alzhrani [7], Wejdan S. Alshehri [8], Nouf Eid Alrashidi [8], Lamya A. Alwahdani [8], Kareem M. Younes [1] and Mohamed S. Refaey [9,10,*]

[1] Department of Pharmaceutical Chemistry, College of Pharmacy, University of Hail, Hail 81442, Saudi Arabia; b.huwaimel@uoh.edu.sa (B.H.); s.alqarni@uoh.edu.sa (S.A.); k.younes@uoh.edu.sa (K.M.Y.)
[2] Medical and Diagnostic Research Centre, University of Hail, Hail 55473, Saudi Arabia; a.alghubyshi@uoh.edu.sa
[3] Department of Biochemistry, Egyptian Drug Authority (Formerly National Organization for Drug Control and Research), Giza 12553, Egypt; marwa10mokhtar1@outlook.com
[4] Department of Pharmaceutical Chemistry, Faculty of Pharmacy, Egyptian Russian University, Cairo 11829, Egypt; mohamed-adel@eru.edu.eg
[5] Drug Design and Discovery Laboratory, Helmy Institute for Medical Sciences, Zewail City of Science and Technology, 6th of October City 12578, Egypt
[6] Department of Clinical Pharmacy, College of Pharmacy, University of Hail, Hail 81442, Saudi Arabia
[7] Department of Pharmaceutics and Industrial Pharmacy, College of Pharmacy, Taif University, P.O. Box 11099, Taif 21944, Saudi Arabia; h.alsaab@tu.edu.sa (H.O.A.); r.zhrani@tu.edu.sa (R.M.A.)
[8] College of Pharmacy, University of Hail, Hail 81442, Saudi Arabia; alseds.wejdan@gmail.com (W.S.A.); alkiari.nouf@gmail.com (N.E.A.); alenazi.lamya@gmail.com (L.A.A.)
[9] Department of Pharmacognosy, Faculty of Pharmacy, University of Sadat City, Sadat City 32897, Egypt
[10] Faculty of Pharmacy, National University of Sadat City, Sadat City 32897, Egypt
* Correspondence: as.ibrahim@uoh.edu.sa (A.S.A.); mohamed.said@fop.usc.edu.eg (M.S.R.)

Content

No.	Contents	Page
**Table S1**	*C. edulis* flower methanolic extract Phytonutrient Profiling.	S3

Table S1. *C. edulis* flower methanolic extract Phytonutrient Profiling.

No.	RT	Compound name	Area %	MF	Molecular formula	Molecular weight
1	4.02	2H-oxecin-2-one,3,4,7,8,9,10-hexahydro-4-hydroxy-10-methyl-,[4S-(4R∗,5E,10S∗)]-	0.41	742	$C_{10}H_{16}O_3$	184
2	6.99	Dotriacontane	0.45	691	$C_{32}H_{66}$	450
3	7.83	9-Octadecenoic acid, (2-phenyl-1,3-dioxolan-4-yl) methyl ester, cis	0.82	640	$C_{28}H_{44}O_4$	444
4	13.26	1,2,3-Propanetriol, 1,2-diacetate	4.27	749	$C_7H_{12}O_5$	176
5	16.34	4H-1-Benzopyran-4-one, 2-(3,4-dimethoxyphenyl)-3,5- dihydroxy-7-methoxy (distinct RT; tentative duplicate flag)	0.76	675	$C_{18}H_{16}O_7$	344
6	17.41	2H-Indeno[1,2-b]furan-2-one, 3,3a,4,5,6,7,8,8b-octahydro-8,8-dime thyl	0.77	701	$C_{13}H_{18}O_2$	206
7	18.89	1H-Cycloprop[e]azulen-7-ol, decahydro-1,1,7-trimethyl-4-methyle ne-, [1ar-(1aα,4aα,7α,7aα,7bα)]	3.21	817	$C_{15}H_{24}O$	220
8	22.07	Mome inositol (monomethylated inositol isomer; specific stereoisomer unconfirmed)	25.10	782	$C_6H_{12}O_6$	194
9	24.55	Oleic Acid	0.74	728	$C_{18}H_{34}O_2$	282
10	24.66	Dodecanoic acid, 2,3-bis(acetyloxy)propyl ester	1.20	724	$C_{19}H_{34}O_6$	358
11	26.36	Cyclopropanebutanoic acid	4.01	751	$C_{25}H_{42}O_2$	374
12	27.27	Hexadecanoic acid	6.68	742	$C_{16}H_{32}O_2$	256
13	27.88	Estra-1,3,5(10)-trien-17α-ol	1.34	776	$C_{18}H_{24}O$	256
14	29.40	4H-1-Benzopyran-4-one, 2-(3,4-dimethoxyphenyl)-3,5- dihydroxy-7-methoxy (distinct RT; tentative duplicate flag)	0.52	713	$C_{18}H_{16}O_7$	344
15	29.49	Cyclopropanebutanoic acid, 2-[[2-[[2-[(2-pentylcyclopropyl)meth yl]cyclopropyl]methyl]cyclopropyl] methyl]-, methyl ester	1.29	775	$C_{25}H_{42}O_2$	374
16	29.63	16-Octadecenoic acid, methyl ester	2.44	756	$C_{19}H_{36}O_2$	296
17	29.77	10-Octadecenoic acid, methyl ester	0.88	690	$C_{19}H_{36}O_2$	296
18	30.15	1,2,4-Trioxolane-2-octanoic acid, 5-octyl-, methyl ester	1.65	690	$C_{19}H_{36}O_5$	344
19	30.53	[1,1'-Bicyclopropyl]-2-octanoic acid, 2'-hexyl-, methyl ester	2.48	830	$C_{21}H_{38}O_2$	322
20	30.66	11-Octadecenoic acid, methyl ester	0.83	722	$C_{19}H_{36}O_2$	296
21	32.31	3,17-Dioxo-11-α-hydroxyand rostane-1,4-diene (tentative/unconfirmed annotation)	1.01	756	$C_{19}H_{24}O_3$	300
22	32.55	Octadecanoic acid, 4-hydroxy-, methyl ester	0.81	718	$C_{19}H_{38}O_3$	314
23	33.0	9,12,15-Octadecatrienoic acid	1.03	706	$C_{27}H_{52}O_4$	496
24	33.18	7-Methyl-*Z*-tetradecen-1-ol acetate	0.88	752	$C_{17}H_{32}O_2$	268
25	33.63	9,12-Octadecadienoic acid (z,z)-,	0.65	678	$C_{27}H_{54}O_4$	498

26	33.91	2-Hydroxy-3-[(9e)-9-octadec enoyloxy]propyl (9e)-9-octadecenoate	0.95	718	$C_{39}H_{72}O_5$	620
27	34.36	Isochiapin B	1.69	746	$C_{19}H_{22}O_6$	346
28	35.16	Spiro[7h-cyclohepta[b]fur an-7,2'(5'h)-furan]-2,5'(3h)-dione	0.98	750	$C_{15}H_{20}O_5$	280
29	35.28	Glycidyl oleate (distinct RT; tentative duplicate flag)	2.33	700	$C_{21}H_{38}O_3$	338
30	35.83	Dotriacontane	2.77	786	$C_{32}H_{66}$	450
31	36.41	3,17-Dioxo-11-α-hydroxyandrostane-1,4-diene (tentative/unconfirmed annotation)	0.80	757	$C_{19}H_{24}O_3$	300
32	36.82	*beta*-Sitosterol	9.37	717	$C_{29}H_{50}O$	414
33	37.04	Stigmast-5-en-3-ol, (3α,24S)-	6.72	748	$C_{29}H_{50}O$	414
34	37.52	oleic acid, 3-(octadecyloxy)propyl ester	2.81	717	$C_{39}H_{76}O_3$	592
35	37.65	Glycidyl oleate (distinct RT; tentative duplicate flag)	0.88	729	$C_{21}H_{38}O_3$	338
36	38.57	Lupeol	3.06	657	$C_{30}H_{50}O$	426

MF: NIST mass spectral library Match factor. Entries with MF < 800 are tentative identifications requiring orthogonal confirmation (e.g., authentic-standard co-injection, chiral analysis, optical rotation, or NMR); entries flagged in the table notes as duplicate or biosynthetically atypical annotations should likewise be treated as tentative.
